# Formation of thioglucoside single crystals by coherent molecular vibrational excitation using a 10-fs laser pulse

**DOI:** 10.1038/s42004-020-0281-6

**Published:** 2020-03-17

**Authors:** Izumi Iwakura, Keiko Komori-Orisaku, Sena Hashimoto, Shoji Akai, Kenta Kimura, Atsushi Yabushita

**Affiliations:** 1 Department of Chemistry, Faculty of Engineering, Kanagawa University, 3-27-1 Rokkakubashi, Yokohama, 221-8686 Japan; 2 Research Institute of Engineering, Kanagawa University, 3-27-1 Rokkakubashi, Yokohama, 221-8686 Japan; 3 Department of Material & Life Chemistry, Faculty of Engineering, Kanagawa University, 3-27-1 Rokkakubashi, Yokohama, 221-8686 Japan

**Keywords:** Chemical physics, Design, synthesis and processing

## Abstract

Compound crystallization is typically achieved from supersaturated solutions over time, through melting, or via sublimation. Here a new method to generate a single crystal of thioglucoside using a sub-10-fs pulse laser is presented. By focusing the laser pulse on a solution in a glass cell, a single crystal is deposited at the edge of the ceiling of the glass cell. This finding contrasts other non-photochemical laser-induced nucleation studies, which report that the nucleation sites are in the solution or at the air-solution interface, implying the present crystallization mechanism is different. Irradiation with the sub-10-fs laser pulse does not heat the solution but excites coherent molecular vibrations that evaporate the solution. Then, the evaporated solution is thought to be deposited on the glass wall. This method can form crystals even from unsaturated solutions, and the formed crystal does not include any solvent, allowing the formation of a pure crystal suitable for structural analysis, even from a minute amount of sample solution.

## Abstract (web summary)

Compound crystallization is typically achieved from supersaturated solutions over time, through melting, or via sublimation. Here the authors show how a 10-fs laser pulse vaporizes a sample of thioglucoside without heating to produce a solvent-free single crystal from an undersaturated solution.

## Introduction

Crystallization of organic compounds is one of the methods for purification and isolation, which are important for various fields such as material function analysis by single crystal structural analysis, drug discovery, and elucidation of life phenomena. The crystallization conditions depend on each compound. Some compounds cannot be crystallized even by changing parameters such as the solvent, density, pH, temperature, and external stimulus. To overcome this limitation, various crystallization methods have been developed.

Laser-induced nucleation by an external physical stimulus has been demonstrated by irradiating an aqueous urea solution with a nanosecond (ns) pulse laser (1.06 μm, 20 ns), in which the nucleation was caused by the optical Kerr effect aligning the urea molecule in the direction of the electric field^1^. This report was followed by various studies of non-photochemical laser-induced nucleation (NPLIN), in which crystallization is induced by the physical stimulus of laser irradiation. This method was reported to enable control of the crystal polymorphism^2–4^. For various compounds, NPLIN has been reported using a ns pulse laser; however, some compounds, such as hen egg-white lysozyme, are not induced to nucleate by the ns laser^5^. Meanwhile, focused irradiation with a femtosecond (fs) pulse laser (800 nm, 120 fs), whose duration is one million times shorter than that of the ns laser, causes cavitation bubbles at the irradiation focal point, which increases the solute density and leads to local nucleation of hen egg-white lysozyme^5^. Crystallization by a fs laser has been widely studied as a promising method to nucleate proteins that have been difficult to crystallize by other conventional methods^6–8^.

In 2007, Sugiyama et al. reported that focused irradiation with a continuous wave (CW) laser (1.06 μm) of a supersaturated solution of glycine led to continuous capture of molecular clusters due to the photon pressure, resulting in the formation of crystal nuclei because of the increase in the molecular density at the focal spot^9^, which is also thought to be another promising method for the formation of crystal nuclei of proteins^10^. This crystallization method using photon pressure (of laser trapping)^11^ has advantages such as the ability to control the time and space of crystallization and crystal growth, to enable crystallization in unsaturated solutions^12^, and to control the crystal polymorphism^9^ and size by tuning the laser polarization and power.

Various mechanisms of NPLIN have been proposed, including the Kerr effect, laser ablation, laser cavitation, photon pressure, and multiphoton absorption, which are still controversial^13^. NPLIN is achievable even for proteins, which are difficult to spontaneously crystallize. However, this method is not applicable to all compounds, and some compounds cannot be crystallized using NPLIN. One example is 2,3,4-tri*-O-*benzyl*-*6*-O-*(N-phenylcarbamoyl)-1-phenylthio-β-D-glucopyranoside (BCPTG, see Supplementary Note 1 and Supplementary Fig. 1). BCPTG has the following characteristics: no absorption band in the visible spectral region and a fine crystalline structure, but formation of a twin crystal by spontaneous crystallization (see Supplementary Note 2). Even at several hundred degrees centigrade and under reduced pressure, vaporization of BCPTG does not proceed, resulting in only carbonization (see the “Methods” section for details). It is known that polymer and sugar compounds are not easily vaporized but are thermally decomposed or denatured by heat, which does not allow the use of crystallization methods such as the melting method or gas-phase method using sublimation. During nucleation and crystal growth in solution of these compounds, the crystal formation is seriously affected by the energy balance of the crystallization conditions (such as the solvent, temperature, solubility, and coexistence in solution), which may cause crystal polymorphism or the formation of poor crystals (polycrystalline structures and twins). Thus, it is necessary to develop methods to deposit a single crystal of compounds for which previous methods cannot be applied.

In the present work, we crystallize BCPTG using visible 10-fs laser pulses^14^, whose duration is much shorter than the traditional femtosecond laser pulse duration of 120 fs. As mentioned above, the fine crystalline structure of BCPTG makes it difficult to produce a single crystal by conventional methods, including NPLIN. One possible breakthrough in the formation of a single crystal of BCPTG is achieved by irradiation with a visible 10-fs laser pulse, as described below. Through this method, a crystal appears at the edge of the ceiling of the glass cell, which is quite different from all the previous NPLIN studies, in which the nucleation site is localized in the solution or at the air–solution interface. The experimental results of the present work show that molecular vibrations are coherently excited by the visible 10-fs laser pulse without heating the sample solution, which converts BCPTG molecules into a gas phase. The crystal is thought to be deposited after the solute is evaporated by excitation of the coherent molecular vibrations. Thus, the finding that irradiation with a 10-fs laser pulse vaporizes BCPTG suggests a novel pathway to produce single crystals while avoiding thermodecomposition.

## Results

### Production of a BCPTG crystal by irradiation with a visible 10-fs laser pulse

**Fig. 1:**
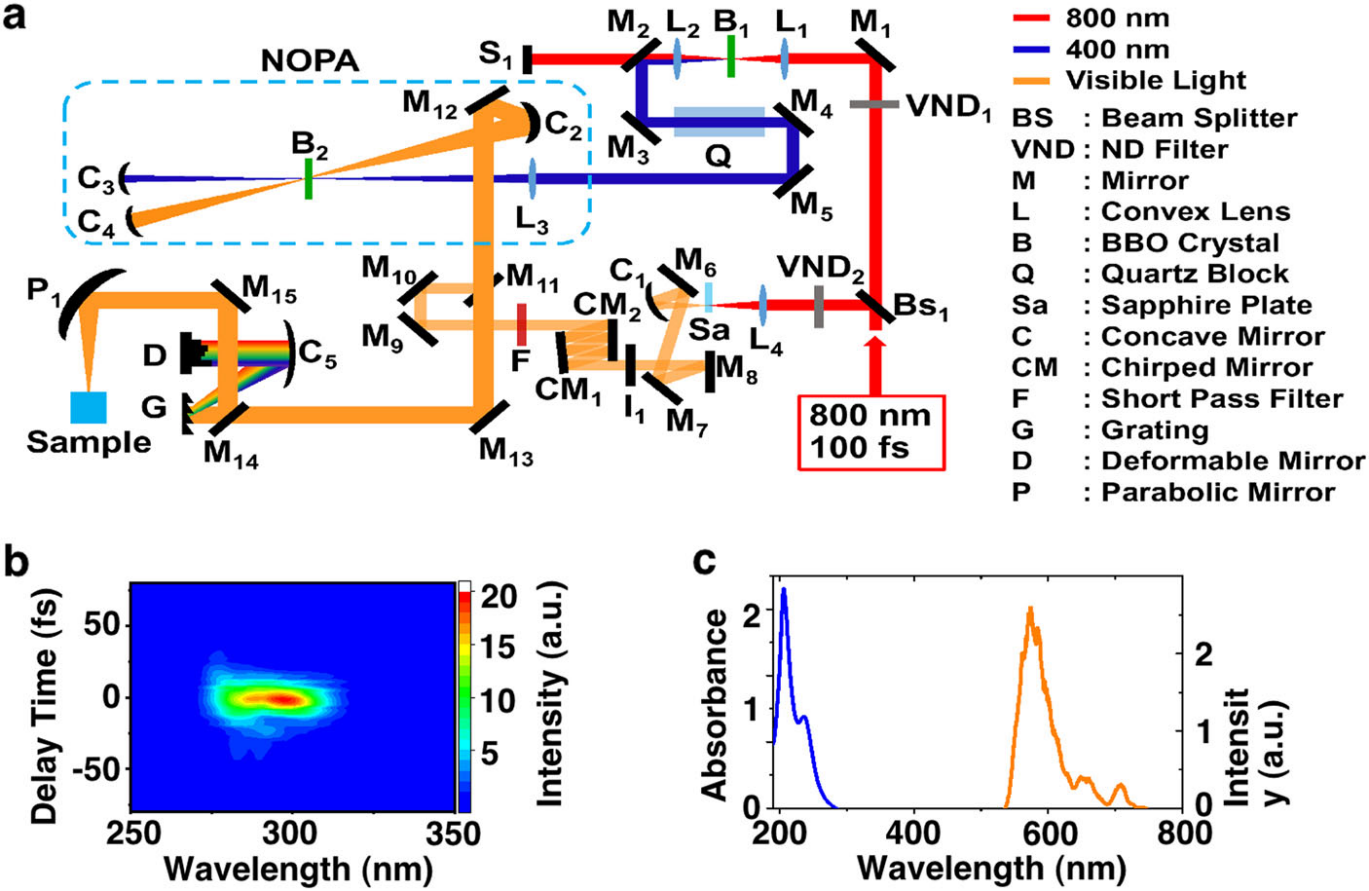
Sub-10-fs visible laser pulses. **a** Apparatus used to generate 10-fs visible laser pulses. **b** Measured FROG trace. **c** Absorption spectrum of BCPTG, and the visible laser spectrum.

**Fig. 2:**
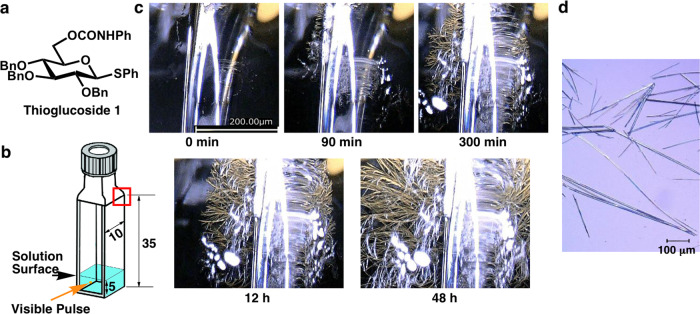
Crystal deposition using 10-fs laser pulses. **a** Molecular structure of 2,3,4-tri*-O-*benzyl*-*6*-O-*(N-phenylcarbamoyl)-1-phenylthio-β-D-glucopyranoside (BCPTG). Bn stands for the benzyl group. **b** Schematic diagram of a sample during laser pulse irradiation (units of mm). The solution was filled to a height of 5 mm from the bottom of the container, and laser pulse irradiation was applied to the solution (at approximately 2 mm below the solution surface). **c** Photographic images showing the state of crystal growth at various time intervals after the start of irradiation. **d** Optical microscopy image of the crystals at 48 h after the start of irradiation shown in **c**.

The 10-fs visible laser pulse (9.5 fs pulse duration, 625 nm center wavelength, 1 kHz repetition rate; see Fig. 1) had an average power of 28 µW (28 nJ pulse^−1^). The laser pulse was focused at a depth of 2 mm below the solution surface (Fig. 2). The focal spot size was 100 μm^2^, which gave a light power density of 28 mJ cm^−2^. After ~90 min of laser irradiation, crystals could be observed by the eye and formed at the top edge of the glass cell approximately 30 mm above the solution surface (Fig. 2c and Supplementary Movie 1). Liquid drops appeared a few millimetres below the crystals and were assumed to be condensed solvent molecules collected on the interior cell surface. Supplementary Movie 1 shows that the solid compounds and solvents evaporated by laser irradiation were cooled at the top of the quartz glass cell such that they transitioned from the gas phase to the solid phase (sublimation) or to the liquid phase. Twelve hours of laser irradiation produced enough crystals for identification by ^1^H-NMR. After 48 h of laser irradiation, the crystal size became large enough for X-ray structural analysis. The deposits obtained after 48 h of laser irradiation were analyzed by optical microscopy and were found to consist of needle-shaped single crystals (see Fig. 2d). The ^1^H-NMR and X-ray analyses confirmed that the produced crystals consisted of BCPTG (see Supplementary Figs. 2 and 3 and Supplementary Tables 1–4). The crystal deposited after 12 h of laser irradiation was analysed by ^1^H-NMR to estimate the required concentration and solvent conditions.

### Dependence on solvent properties of BCPTG crystallization

These experiments were designed and evaluated to determine whether this method works even when the solution concentration is lower than the saturation concentration. For solution concentrations of 0.4, 0.8, 1.1, and 1.5 mM (0.26, 0.53, 0.73, and 0.99 g L^−1^), no difference was found in the deposited morphology of the needle-shaped crystals. However, a solution concentration of 0.2 mM (0.13 g L^−1^) did not lead to the deposition of any crystals after 12 h of irradiation (no crystal was deposited even after 24 h of irradiation).

In addition, the dependence of this method on the solvent properties was studied. A control experiment was performed using solvents with comparable dielectric constants (acetonitrile and acetone) and solvents with smaller dielectric constants than those of the alcohols. The experiment was repeated using different solvents (see Table 1)^15–17^. The solvents tetradeuteromethanol, ethanol, 2-propanol, and acetonitrile all yielded crystals at the top edge of the glass cell. However, no crystals formed when using acetone, chloroform, benzene, toluene, 1,4-dioxane, or cyclooctanol as the solvent.

**Table 1:** Physicochemical properties of the solvents and experimental conditions when used for crystallization of BCPTG.

Solvent	Dielectric constant^15,16^	Viscosity (mPa⋅s)^a^^16,17^	Boiling point (degree)	Saturation concentration (mM)	Solution concentration (mM)	Crystal deposited
Methanol	32.63^a^	0.54		1.7	1.5	√
Tetradeuteromethanol			65.4		1.5	√
Ethanol	24.3^a^	1.07	78	2.2	1.5	√
2-Propanol	18.3^a^	2.04	97	1.1	1.0	√
Cyclooctanol	11.8^a^	149	106–108^c^	1.5	1.0	–
Acetonitrile	37.5^b^	0.37	82	5.5	1.5	√
Acetone	20.7^a^	0.31	56	150	1.5	–
Chloroform	4.806^b^	0.54	61	>600	1.5	–
Benzene	2.274^a^	0.60	80	115	1.5	–
Toluene	2.379^a^	0.56	110.6	45	1.5	–
1,4-Dioxane	2.209^a^	1.18	100–102	>300	1.5	–

^a^25 °C.

^b^20 °C.

^c^22 mm Hg.

## Discussion

The crystals produced in the present work were confirmed to be BCPTG crystals by ^1^H-NMR and X-ray analyses. Each factor of “the wettability of the quartz glass cell affecting the nucleation rate”, “solvent evaporation of invisible tiny solution droplets attached to the glass wall dispersed by ablation”, and “NPLIN” may affect the crystallization process of the present work; however, their contributions were refuted, as shown in the following (a detailed discussion can be found in the Supplementary Discussion).

Even when the quartz cell was silyl coated, a crystal was also deposited at the edge of the ceiling of the glass cell (see Supplementary Fig. 4). When we used a quartz glass cell with a glass rod pointing downward from the center of the top plastic screw cap, with the tip located at a height 8 mm above the solution surface (Supplementary Fig. 5a), irradiation with the 10-fs laser pulse produced a droplet on the glass rod, and then, a BCPTG crystal deposited there (Supplementary Figs 5b, c and 6). Both of these results indicate that the wettability of the quartz glass cell does not cause the crystallization.

We used a glass cell with a height of 100 mm (2.5 times that of the original cell), which still led to a crystal being produced at the edge of the ceiling of the glass cell (Supplementary Fig. 7). The depth of the irradiation point from the liquid level and the irradiation power did not affect the crystallization result. These results show that laser ablation does not contribute to the present crystallization phenomena. The required irradiation energy of the present method also refutes the contribution of laser ablation as follows. The ablation threshold of organic compounds^18^ is reported to be 5–30 mJ cm^−2^. Meanwhile, in the present work, a crystal was deposited even with an irradiation energy of 0.5 mJ cm^−2^ (1/10 of that used in ref. ^18^).

Compared with the NPLIN previously reported, the following differences can be found. In those NPLIN studies, the nucleation sites were in the solution or at the air-solution interface. However, in the present work, the crystal was deposited at the edge of the ceiling of the glass cell. In those NPLIN studies, the induction time was always short (from 1 to 30 min) for when the crystal could be observed by microscopy. Meanwhile, the present method required irradiation for as long as 90 min until crystals could be seen by the eye. The differences in the crystal deposition sites refute the crystallization method using photon pressure (of laser trapping).

We irradiated a nanosecond Nd:YAG laser pulse (532 nm, 10 ns, 2.6 J cm^−2^) for 12 h, which did not lead to the deposition of any crystals. This confirms that the effect of the optical Kerr effect is negligible for the visible pulse irradiation on this sample.

The energy of the visible 10-fs laser pulse used in the present work was 28 nJ pulse^−1^ (28 mJ cm^−2^), which is much lower than the threshold of cavitation^6^ previously reported, 0.4–4 μJ pulse^−1^ (80–400 J cm^−2^) for a 200-fs laser pulse and 0.9–8 μJ pulse^−1^ (180–1620 J cm^−2^) for a 1800-fs laser pulse. Thus, it is thought that cavitation bubbles were not produced under the present conditions.

Therefore, the present crystallization phenomena cannot be explained by the previous NPLIN mechanism, which led us to consider a novel mechanism based on phase transformation induced by reaction via coherent Raman vibration excitation. The following discussion shows that the experimental results support this novel mechanism.

The 10-fs visible laser pulse (see Fig. 1) has a broad bandwidth of 5255 cm^−1^ extending from 525 to 725 nm, i.e., 19048–13793 cm^−1^ (see Fig. 1c), which can coherently excite a wide range of Raman-active vibrational modes (vibrational modes whose excitation is allowed by the symmetry selection rules of stimulated Raman processes) under impulsive excitation within the 10-fs pulse duration. In our previous work, we applied this coherent vibrational excitation to trigger the reaction in the electronic ground state^19–21^, which is summarized in the following section.

Coherent molecular vibrations were found to trigger reactions in the electronic ground state, such as thermal reactions, which is called “reaction by coherent molecular vibration (RCMV)”^19–21^. The thermal energy of 5255 cm^−1^ corresponds to a temperature of 7500 K considering *E* = *kT*, where *E*, *k*, and *T* represent the energy, Boltzmann constant, and absolute temperature, respectively. The broad bandwidth of the laser can excite high-energy vibrational levels that require temperatures of up to 7500 K when obtained through thermal excitation to overcome the activation energy necessary to trigger thermal reaction. Thus, irradiation with the 10-fs visible laser can induce RCMV without heating the samples to high temperatures.

The sample solution in this study has no absorption at wavelengths longer than 290 nm and thus cannot be excited into an electronic excited state by single-photon absorption under the 10-fs visible laser pulse. Therefore, irradiation with the 10-fs visible laser pulse was expected to excite coherent molecular vibrations in the electronic ground state. Note that the photoreaction proceeds in parallel with RCMV if a sample is irradiated by an ultrashort laser pulse whose photon energy is higher than the minimum transition energy, Δ*E*_min_, of the sample^20,22^. To study RCMV (while avoiding photoreaction), it is necessary to excite a sample by an ultrashort pulse laser whose photon energy is lower than the Δ*E*_min_ of the sample.

In the present work, to form a single crystal, irradiation with the 10-fs laser pulse is thought to excite the coherent molecular vibrations, which was confirmed as follows.

The molecular vibration of the solute and solvent coherently excited by the 10-fs visible laser pulse was studied as follows. The methanol solution of BCPTG irradiated by the 10-fs visible laser pulse was probed by the same laser pulse to perform pump–probe measurements to study the vibrational dynamics (the measurement scheme is briefly described in the “Methods” section). The pulse duration of 10 fs is much shorter than the vibrational period, which allows visualization of the signal modulation reflecting the vibrational period. The measured transient absorption shows modulation of the period of the molecular vibration, indicating that the wavepacket was coherently excited by the pulsed laser irradiation. Observation of the time dependence of the signal modulation elucidates the ultrafast dynamics of the molecular vibration.

**Fig. 3:**
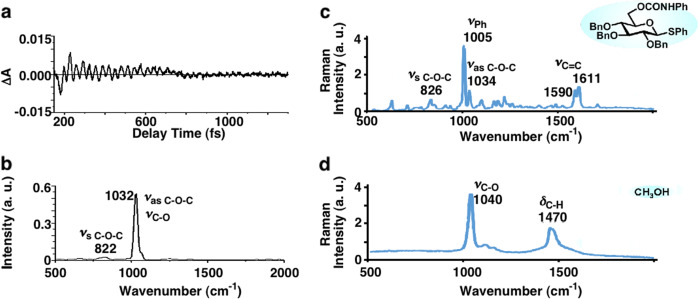
Results of pump–probe measurements of the BCPTG methanol solution. **a** Real-time trace. **b** Fast Fourier transform power spectrum. Raman spectra of **c** BCPTG and **d** methanol.

The measured trace of the absorption change, Δ*A*, shows a periodic vibration at approximately Δ*A* = 0 (see Fig. 3a), which does not show decay upon relaxation from the electronic excited state to the electronic ground state. This finding is thought to be reasonable because, considering that the photon energy of the 10-fs laser pulse (19,048–13,793 cm^−1^) is much lower than the minimum electronic transition energy (>34,500 cm^−1^), the observed signal modulation is thought to reflect the molecular vibration in the electronic ground state^19–21^. The Fourier transform spectrum of ΔA, plotted in Fig. 3b, shows two peaks that are thought to correspond to the Raman spectrum, although the signal intensity of the Fourier spectrum can be different from that of the Raman spectrum due to the effect of the Raman cross section. Based on a comparison with the Raman spectra of BCPTG and methanol, shown in Fig. 3c, d, the two peaks observed in the Fourier spectrum can be assigned as follows. The peak at 1032 cm^−1^ is assigned to the C–O–C asymmetric stretching mode of BCPTG and the C–O stretching mode of methanol. This broad peak may also include the signal of the symmetric ring stretching mode of the phenyl group with a frequency of 1005 cm^−1^. The peak at 822 cm^−1^ is assigned to the C–O–C symmetric stretching mode of BCPTG. These results suggest that Raman-active modes were excited in both BCPTG and the solvent. These Raman modes were assigned according to the literature^23,24^. Thus, it has been confirmed that the Raman-active vibrational modes of BCPTG and the solvent were coherently excited under irradiation with the 10-fs visible laser pulse.

Conventional thermal evaporation does not occur in BCPTG because the boiling temperature is higher than the temperature of carbonization; however, irradiation with the 10-fs visible laser pulse may induce a physical phase transformation from the solution phase into the gas phase (see Supplementary Movie 1). After irradiation with the visible 10-fs pulse laser for 12 h, the sample cell was left to stand for one day, and the amount of the deposited sample increased. This result indicates that BCPTG was floating in the gas phase. We hypothesized that the crystal was deposited from a gas-phase product of the physical phase transformations triggered by coherent Raman-active molecular vibrational excitations.

**Fig. 4:**
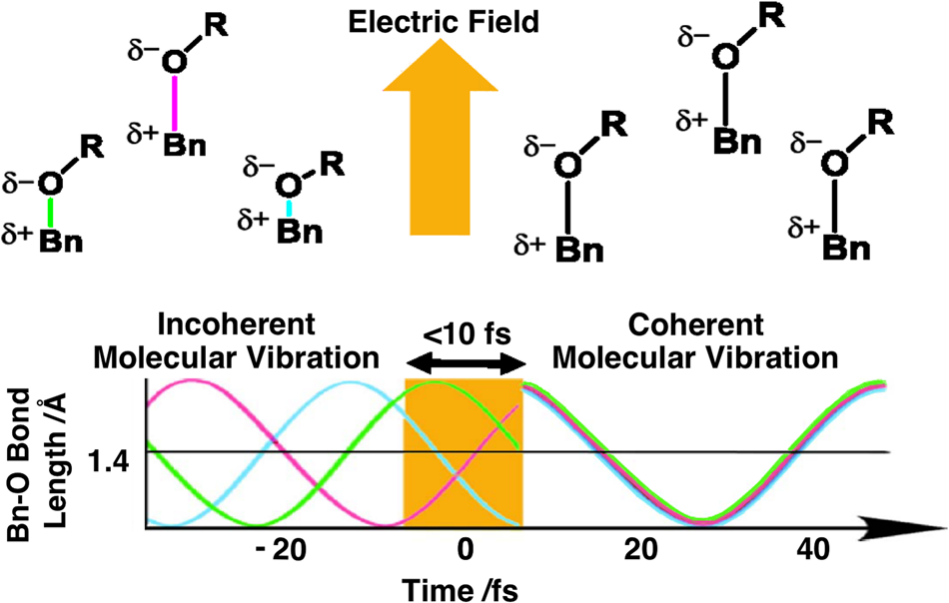
Diagram summarizing the coherent molecular vibrational excitation process. The BCPTG molecule is modelled as Bn-O-R. For each Bn-O bond shown by different colors, the time dependence of the bond length is plotted as a curve using the same color corresponding to each Bn-O bond. Before irradiation with the sub-10-fs laser pulse (in the negative delay region), the phases of the Bn-O stretch are random. Around zero delay (yellow coloured region in the figure), the molecules were irradiated with the sub-10-fs laser pulse, which led to the phases of the Bn-O stretch being the same for the irradiated molecules.

The vaporization mechanism via coherent excitation of the Raman-active vibrational modes of BCPTG and the solvent can be explained as follows. Irradiation with the broadband laser pulse excites various Raman-active molecular vibrational modes in both BCPTG and the solvent (Fig. 3c, d). The electric field of the laser beam is vertically polarized and has a high peak intensity, oscillating for a few cycles within the 10-fs pulse duration. The interaction between the intense optical electric field and molecular polarization via the induced Raman process allows the pulse irradiation to coherently excite molecular vibrations (Fig. 4), thus moving the molecules from the solution phase into the gas phase. This suggests that the vaporization is independent of the direction of the linear polarization (see Supplementary Tables 5 and 6). This coherent Raman-active molecular vibration lowers the energy required for vaporization of BCPTG and the solvent, and both compounds can enter the gas phase at ambient temperature and pressure. The following discussion provides a further analysis of the conditions required for physical phase transformation (vaporization) by coherent molecular vibration.

One possible mechanism causing the observed difference in different solvents is coherent molecular vibration. The electric field of the laser pulse can interact with polarized chemical bonds. Solvents with lower dielectric constants (chloroform, benzene, toluene, and 1,4-dioxane) have chemical bonds that are less polarizable, which hinders their interaction with the electric field of the laser pulse, resulting in lower efficiency in exciting coherent molecular vibrations. Solvents with high dielectric constants have effectively excited coherent Raman-active molecular vibrations, allowing evaporation and crystal deposition of BCPTG. Note that a polarizable chemical bond exists in acetone, but it is not Raman active; thus, coherent molecular vibrations cannot be excited when acetone is used as the solvent.

Another possible origin of the differences in the crystal deposition behavior is the difference in the solubility limits. It was proposed based on the observed results that crystals are not formed in solvents with a high saturation concentration (>10 mM) but are formed (except for in cyclooctanol) in solvents with a low saturation concentration (1.0–1.5 mM). When we use a solution with a higher saturation concentration, a BCPTG crystal may still be deposited, but the nucleus of the deposited BCPTG is redissolved by the liquid solvent. The reason why cyclooctanol does not lead to crystal deposition is thought to be that its high viscosity^16,17^ prevents vaporized BCPTG from leaving the liquid (it is generally known that a higher viscosity results in a higher boiling point).

Thus, these results for the comparison of cases using different solvents are contingent on the two main physicochemical properties of the solvents (the coherent molecular vibration and the saturation concentration) discussed as possible mechanisms, which will be followed up on in our future work on redundancies to study the stochastic effect of this nucleation mechanism.

**Fig. 5:**
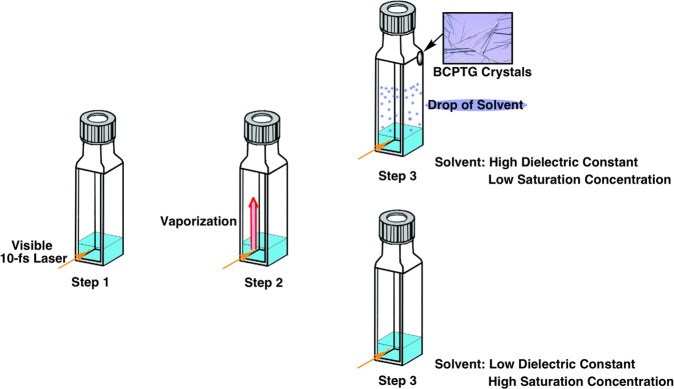
Schematic of the proposed crystallization mechanism using a 10-fs laser pulse. In step 1, the unsaturated solution is irradiated with the short laser pulse, in step 2 vaporization occurs. In step 3, the vapor deposits to form crystals. Saturation of the sample solution is not required in this method.

Until now, vaporizing a molecule as large as BCPTG (i.e., with a molar mass of 661.8 g) under ambient conditions was believed to be impossible. Our present work demonstrates that this transition can be accomplished by irradiating a solution with 10-fs visible laser pulses. A schematic of the mechanism is shown in Fig. 5. Using a wavelength that does not cause photoexcitation, Raman-active vibrational modes of the solute and solvent are coherently excited to cause physical phase transformations (evaporation). Then, a transition from the gas phase to the solid phase (sublimation) occurs when the sample is cooled at the top of the glass cell, producing single BCPTG crystals without carbonization by thermodecomposition. A crystal was deposited at the top of the glass cell independent of the glass cell shape (see Supplementary Fig. 8). The fact that crystals are concentrated at the corner is probably due to the fluid mechanism of the gas in the sample holder.

Most crystallization methods for BCPTG tend to produce a twin crystal; thus, producing single crystals by these methods is challenging. The non-twinned crystal produced in the present method is thought to be due to the possible 2-D crystal growth. In this system, since the solute concentration in the gas phase is low, the perpendicular growth of the flat faces is considered to grow via spiral growth or two-dimensional nucleation and growth^25,26^. Therefore, the proportion of twins which grow via rough growth mechanism is low. The twin crystal contains single crystals growing in different directions, which hinders the ability to perform X-ray crystal structural analysis because of the increase in the number of parameters. Therefore, it is preferred to prepare a single crystal for X-ray crystal structural analysis. By developing a novel method to generate a single crystal, it becomes possible to perform X-ray crystal structural analysis, even for crystals that could not be obtained or were difficult to obtain by traditional crystallization methods. Our present work demonstrates that single crystals can be obtained by irradiation with a 10-fs laser pulse to excite coherent molecular vibrations.

Although our present method requires Raman-active polarized atomic bonds, this method has a specific advantage in that it does not require thermal excitation to produce the crystal. Using this method, the produced crystal is not solvated. Thus, the present scheme is a practical, revolutionary method to produce single crystals, even those that cannot be obtained by traditional crystallization methods.

## Methods

### Laser experimental setup

A schematic diagram of the laser pulse generation system is shown in Fig. 1a. A Ti:sapphire regenerative amplifier (SpectraPhysics, Spitfire Pro) pulse laser produced a near-infrared (NIR) laser pulse (pulse duration of 100 fs, center wavelength of 800 nm, repetition rate of 1 kHz) that was separated into two laser pulses by a beam splitter. One of the pulses was focused onto a nonlinear crystal to generate a second harmonic (SH), which was used as a pump pulse in the subsequent amplification process. The other pulse was focused onto a sapphire plate to broaden its spectral bandwidth into femtosecond visible white light (500–750 nm). The pulse duration of the visible white pulse was compressed using a chirped mirror pair and then amplified over the entire visible broadband region using noncollinear optical parametric amplification (NOPA)^14,27^. The duration of the amplified pulse was compressed using a diffraction grating (G) and a deformable mirror (D). The pulse was focused into a sample solution stored in a quartz glass cell. The ultrafast dynamics of the sample solutions were measured at the focal point. To estimate the pulse duration after transmission through the front wall of the quartz glass cell, pulse characterization was performed by inserting a quartz glass plate with the same thickness as the front wall of the quartz glass cell (1 mm) into the optical path (between M_13_ and M_14_). The compressed pulse duration was estimated to be 9.5 fs using the frequency-resolved optical gating (FROG) method (see Fig. 1b). The spectrum of the 10-fs visible laser pulse ranged from 525 to 725 nm (see Fig. 1c). After the pulse characterization was complete, the inserted quartz glass plate was removed before initiating crystallization by 10-fs laser pulse irradiation and measuring the ultrafast dynamics of the sample solutions.

### Sample

The chemical synthesis procedures of the samples are given in the Supplementary Methods along with the NMR spectra (Supplementary Figs. 9–15) and physical measurements. The solubility curve of BCPTG in methanol is shown in Supplementary Fig. 16. Conventional thermal evaporation of BCPTG does not occur with increasing temperature; instead, carbonization by thermodecomposition is observed above 200 °C. In addition, BCPTG does not evaporate under a reduced pressure (20 hPa), whereas BCPTG undergoes thermodecomposition at temperatures above 190 °C and never evaporates at any temperature. Thus, BCPTG cannot be vaporized under conventional heating processes. We confirmed that BCPTG cannot be vaporized at normal pressure or at a reduced pressure (20 hPa).

### Laser pulse irradiation

The obtained crystals of Compound 1 (BCPTG) were recrystallized from methanol before use. The BCPTG crystal was dissolved in methanol to a saturation concentration of 1.7 mM. To avoid precipitation of crystals, the solution was diluted with methanol to 1.5 mM to obtain an “almost saturated solution”. This solution was filtered through a syringe filter with a 0.45 µm hydrophobic PTFE membrane. A quartz glass cell (wall thickness of 1 mm, internal dimensions of 10 × 10 × 40 mm^3^) was washed several times with acetone followed by methanol and dried before each irradiation trial. The solution with a volume of 0.5 mL was carefully placed using a syringe, to ensure no splashing of the solution, at the bottom of the sample cell. The cell containing the solution was closed with a screw cap and sealed with PARAFILM tape (laboratory film). Then, the sample was irradiated with the laser by placing the sample as shown in Fig. 1a. The crystallization experiment was performed at room temperature (22 ± 0.5 °C) under ambient pressure. The Supplementary Methods section gives details of the experiments performed to study the dependence of the crystallization on the polarization of the irradiated light (Supplementary Fig. 17) and on the glass cell shape. Data for confirmation of the reproducibility are given in Supplementary Methods and Supplementary Tables 7–10.

### Pump–probe measurement

For pump–probe measurements, the optical system was changed as shown in Supplementary Methods and Supplementary Fig. 18. The broadband 10-fs visible laser pulse was separated into two pulses using a beam sampler (BS_2_). The two pulses had the same spectrum and pulse duration but different powers with a power ratio of 10:1. The pulses with higher and lower intensities were used as the pump and probe pulses, respectively. A pulse compressor system was inserted before the beam sampler so that the pulse duration of the two pulses after passing through the front glass wall of the sample quartz glass cell was 10 fs. The retroreflector inserted in the optical path of the pump pulse was moved to scan the optical delay between the pump pulse and the probe pulse on the sample. The optical delay was scanned from −100 fs to 1200 fs with a 1-fs step. For each optical delay, we measured the absorption change in the probe pulse caused by the pump pulse irradiation. Similar to the crystallization experiment, the pump–probe measurement was also performed at room temperature (22 ± 0.5 °C) under ambient pressure. For the pump–probe measurement, 0.4 mL of the solution (1.5 mM) was placed in a quartz glass cell with an optical path length of 1 mm (internal dimensions of 10 × 1 × 40 mm^3^) and a wall thickness of 1 mm, which was sealed with a screw cap prior to laser pulse irradiation.

## Supplementary information

Supplementary Information

Description of Additional Supplementary Files

Supplementary Data 1

Supplementary Movie 1

## Data Availability

The data supporting the findings of this study are available within the article and its Supplementary Information files (NMR Spectra, X-ray Structure, Supplementary Discussion, Supplementary Methods). All other relevant source data are available from the corresponding author upon reasonable request. The X-ray crystallographic coordinates for the structures reported in this study have been deposited at the Cambridge Crystallographic Data Centre (CCDC), under deposition number 1834656. These data can be obtained free of charge from The Cambridge Crystallographic Data Centre via http://www.ccdc.cam.ac.uk/data_request/cif.
