# Supplementary material for: Formation of thioglucoside single crystals by coherent molecular vibrational excitation using a 10-fs laser pulse
Source: Commun Chem. 2020 Mar 17;3:35. doi: 10.1038/s42004-020-0281-6 (PMC9814847; doi:10.1038/s42004-020-0281-6)
Supplement: Supplementary file 1 — Supplementary Information [file 42004_2020_281_MOESM1_ESM.pdf]

Supplementary Information

Formation of thioglucoside single crystals by coherent molecular vibrational excitation using a 10-fs laser pulse

Izumi Iwakura et al.,

Supplementary Note 1. Verification for nucleation of BCPTG on irradiation of 800-nm 100-fs pulse laser

Pulse laser from Ti:sapphire regenerative amplifier (wavelength 800 nm, pulse duration 100 fs, repetition rate 1 kHz) was focused into the sample solution using a 10x objective lens. The focus spot is set on the back surface of the glass cell. The light intensity was set to be 0.12 μJ pulse^{-1} (24 J cm^{-2}) which is slightly lower than the threshold to generate bubble at the light focus spot. Irradiation has continued for six hours; however, nucleation of crystal could not be observed.

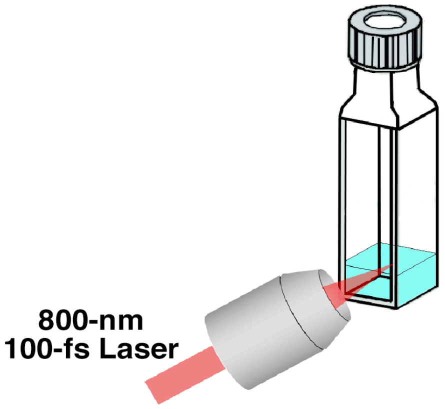

Supplementary Figure 1. **Schematic figure of the experimental setup using 100-fs pulse laser.**

Supplementary Note 2. Twin Crystal

Single crystal structure analysis was performed on crystals appeared from methanol solution. The Flack parameter of the analysis data is shown below.

Flack x = 0.422(239) from 292 selected quotients (Parsons' method)

The value of Flack parameter indicates that the crystal has an inversion twin component[1,2].

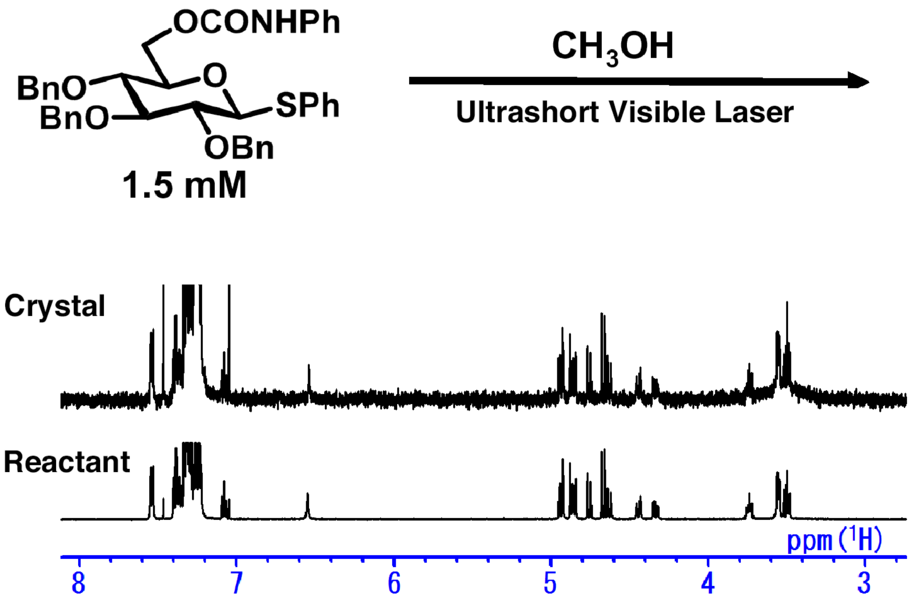

Supplementary Figure 2. **^{1}H-NMR spectra of the reactant and crystals sublimed from a methanol solution of BCPTG.**

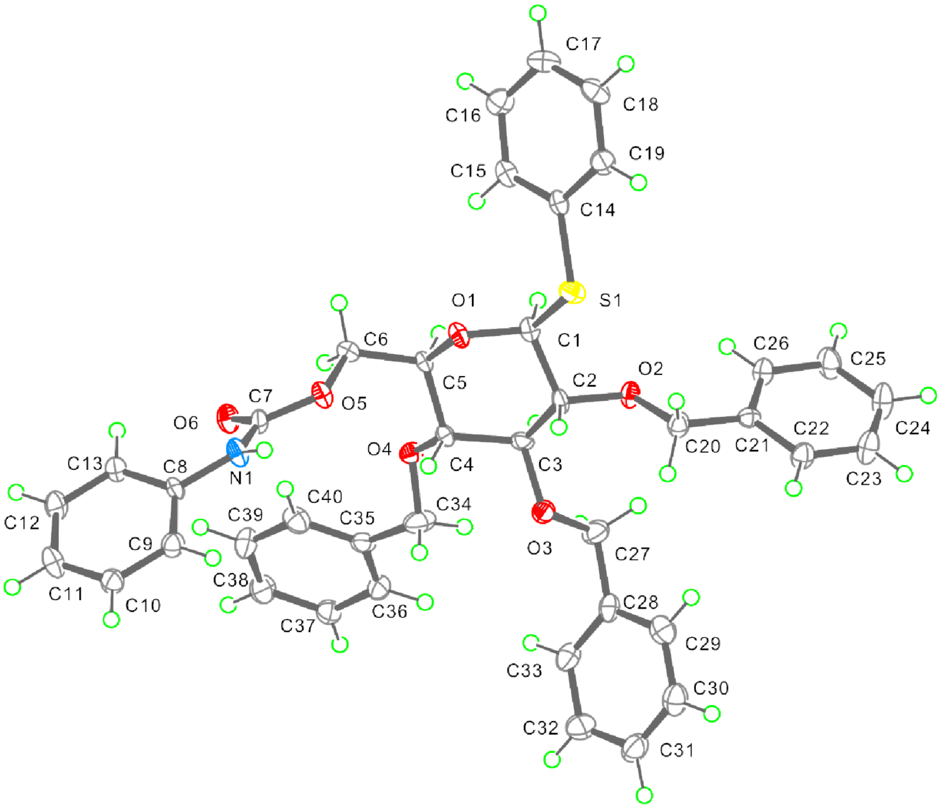

Supplementary Figure 3. The crystal structure of 2,3,4-tri-*O*-benzyl-6-*O*-(N-phenylcarbamoyl)-1-phenylthio-β-D-glucopyranoside (BCPTG). Displacement ellipsoids are drawn at the 50% probability level. Gray, blue, red, yellow and green spheres represent C, N, O, S and H atoms, respectively.

Supplementary Table 1. Crystal Data and Refinement Parameters of BCPTG.

**Crystal data**	Chemical formula	$C_{40}H_{39}NO_6S$
	M_r	661.78
	Crystal system	Monoclinic
	Space group	$P2_1$
	Temperature (K)	93
	a, *b*, *c* (Å)	16.164 (7), 5.351 (2), 19.099 (8)
	β (°)	93.062 (5)
	V (Å^3)	1649.6 (12)
	Z	2
	Radiation type	Mo *K*α
	μ (mm^{-1})	0.149
	Crystal size (mm)	0.58 × 0.03 × 0.01
**Data collection**	Diffractometer	Rigaku Saturn724
	Absorption correction	Numerical
		NUMABS (Rigaku, 1999)
	T_{min}, T_{max}	0.995, 0.999
	No. of measured	13454
	No. of independent	7415
	No. of observed [$I > 2\sigma(I)$]	4437
	R_{int}	0.0627
**Refinement**	$R[F^2 > 2\sigma(F^2)]$	0.0645
	$wR(F^2)$	0.1346
	S	1.053
	No. of reflections	7415
	No. of parameters	433
	No. of restraints	1
	H-atom treatment	H-atom parameters constrained
	$\Delta\rho_{max}$, $\Delta\rho_{min}$ (e Å^{-3})	0.29, −0.34
	Frack parameters	0.10 (10) / 1232 Friedel pairs

Supplementary Table 2. Selected bond distances (Å).

S1	-	C1	1.797(5)	S1	-	C14	1.765(6)
O1	-	C1	1.406(6)	O4	-	C34	1.419(6)
O1	-	C5	1.435(6)	O4	-	C4	1.441(6)
O2	-	C2	1.426(6)	O5	-	C6	1.450(6)
O2	-	C20	1.417(6)	O5	-	C7	1.348(6)
O3	-	C27	1.441(6)	O6	-	C7	1.218(7)
O3	-	C3	1.440(6)		-		
N1	-	C7	1.361(7)	N1	-	C8	1.417(6)
C1	-	C2	1.534(7)	C21	-	C26	1.385(7)
C2	-	C3	1.529(7)	C22	-	C23	1.370(7)
C3	-	C4	1.506(7)	C23	-	C24	1.374(8)
C4	-	C5	1.524(6)	C24	-	C25	1.394(8)
C5	-	C6	1.503(7)	C25	-	C26	1.377(7)
C8	-	C13	1.385(7)	C27	-	C28	1.518(7)
C8	-	C9	1.395(7)	C28	-	C29	1.390(8)
C9	-	C10	1.389(7)	C28	-	C33	1.381(8)
C10	-	C11	1.388(8)	C29	-	C30	1.387(8)
C11	-	C12	1.386(8)	C30	-	C31	1.382(8)
C12	-	C13	1.382(7)	C31	-	C32	1.380(8)
C14	-	C15	1.386(7)	C32	-	C33	1.393(8)
C14	-	C19	1.382(7)	C34	-	C35	1.502(7)
C15	-	C16	1.398(8)	C35	-	C36	1.373(8)
C16	-	C17	1.390(8)	C35	-	C40	1.381(7)
C17	-	C18	1.369(8)	C36	-	C37	1.389(7)
C18	-	C19	1.385(7)	C37	-	C38	1.369(8)
C20	-	C21	1.500(7)	C38	-	C39	1.381(8)
C21	-	C22	1.391(7)	C39	-	C40	1.397(8)

Supplementary Table 3. **Selected bond angles (deg).**

C14	-	S1	-	C1	105.5(3)		C19	-	C14	-	C15	120.1(5)
C1	-	O1	-	C5	111.1(4)		C19	-	C14	-	S1	115.5(4)
C20	-	O2	-	C2	114.8(4)		C15	-	C14	-	S1	124.3(4)
C3	-	O3	-	C27	115.2(4)		C14	-	C15	-	C16	118.6(5)
C34	-	O4	-	C4	115.4(4)		C17	-	C16	-	C15	120.9(6)
C7	-	O5	-	C6	115.5(4)		C18	-	C17	-	C16	119.5(5)
C7	-	N1	-	C8	124.5(4)		C17	-	C18	-	C19	120.1(5)
							C14	-	C19	-	C18	120.6(5)
O1	-	C1	-	C2	109.8(4)		O2	-	C20	-	C21	109.7(5)
O1	-	C1	-	S1	110.9(4)		C26	-	C21	-	C20	121.5(5)
C2	-	C1	-	S1	107.6(4)		C22	-	C21	-	C20	119.5(5)
O2	-	C2	-	C1	109.8(4)		C26	-	C21	-	C22	118.9(5)
C3	-	C2	-	C1	112.1(4)		C23	-	C22	-	C21	120.3(5)
O2	-	C2	-	C3	107.8(4)		C22	-	C23	-	C24	121.1(6)
O3	-	C3	-	C2	107.7(4)		C23	-	C24	-	C25	118.9(6)
C4	-	C3	-	C2	113.5(4)		C26	-	C25	-	C24	120.3(6)
O3	-	C3	-	C4	110.6(4)		C25	-	C26	-	C21	120.4(5)
O4	-	C4	-	C3	112.2(4)		O3	-	C27	-	C28	107.6(4)
O4	-	C4	-	C5	104.1(4)		C33	-	C28	-	C27	120.8(5)
C3	-	C4	-	C5	110.6(4)		C29	-	C28	-	C27	120.8(5)
O1	-	C5	-	C4	110.7(4)		C33	-	C28	-	C29	118.4(6)
C6	-	C5	-	C4	114.4(4)		C30	-	C29	-	C28	120.7(6)
O1	-	C5	-	C6	109.1(4)		C31	-	C30	-	C29	120.5(6)
O5	-	C6	-	C5	110.4(4)		C32	-	C31	-	C30	119.3(6)
O6	-	C7	-	N1	126.0(5)		C31	-	C32	-	C33	120.1(6)
O5	-	C7	-	N1	109.1(5)		C28	-	C33	-	C32	121.1(6)
O6	-	C7	-	O5	124.9(5)		O4	-	C34	-	C35	111.5(5)
C13	-	C8	-	C9	119.5(5)		C36	-	C35	-	C34	117.9(5)
C13	-	C8	-	N1	122.3(5)		C40	-	C35	-	C34	122.3(5)
C9	-	C8	-	N1	118.2(5)		C36	-	C35	-	C40	119.7(5)
C10	-	C9	-	C8	120.3(5)		C35	-	C36	-	C37	121.1(6)
C11	-	C10	-	C9	120.1(5)		C38	-	C37	-	C36	119.2(6)
C12	-	C11	-	C10	119.1(5)		C37	-	C38	-	C39	120.5(6)
C13	-	C12	-	C11	121.3(5)		C38	-	C39	-	C40	120.0(6)
C12	-	C13	-	C8	119.7(5)		C35	-	C40	-	C39	119.4(5)

Supplementary Table 4. **Selected torsion angles (deg).**

C5	-	O1	-	C1	-	C2	-64.3(5)		N1	-	C8	-	C13	-	C12	-179.0(5)
C5	-	O1	-	C1	-	S1	176.9(3)		C1	-	S1	-	C14	-	C19	-161.7(4)
C14	-	S1	-	C1	-	O1	-80.5(4)		C1	-	S1	-	C14	-	C15	20.8(5)
C14	-	S1	-	C1	-	C2	159.5(3)		C19	-	C14	-	C15	-	C16	0.0(8)
C20	-	O2	-	C2	-	C3	-119.6(5)		S1	-	C14	-	C15	-	C16	177.4(4)
C20	-	O2	-	C2	-	C1	118.1(5)		C14	-	C15	-	C16	-	C17	-1.2(8)
O1	-	C1	-	C2	-	O2	172.1(4)		C15	-	C16	-	C17	-	C18	1.9(9)
S1	-	C1	-	C2	-	O2	-67.1(5)		C16	-	C17	-	C18	-	C19	-1.5(9)
O1	-	C1	-	C2	-	C3	52.3(6)		C15	-	C14	-	C19	-	C18	0.3(8)
S1	-	C1	-	C2	-	C3	173.1(4)		S1	-	C14	-	C19	-	C18	-177.2(4)
C27	-	O3	-	C3	-	C4	137.1(5)		C17	-	C18	-	C19	-	C14	0.4(9)
C27	-	O3	-	C3	-	C2	-98.3(5)		C2	-	O2	-	C20	-	C21	177.5(4)
O2	-	C2	-	C3	-	O3	72.8(5)		O2	-	C20	-	C21	-	C26	16.3(7)
C1	-	C2	-	C3	-	O3	-166.3(4)		O2	-	C20	-	C21	-	C22	-165.6(5)
O2	-	C2	-	C3	-	C4	-164.5(4)		C26	-	C21	-	C22	-	C23	1.7(8)
C1	-	C2	-	C3	-	C4	-43.5(6)		C20	-	C21	-	C22	-	C23	-176.4(5)
C34	-	O4	-	C4	-	C3	64.4(6)		C21	-	C22	-	C23	-	C24	-0.3(9)
C34	-	O4	-	C4	-	C5	-176.0(4)		C22	-	C23	-	C24	-	C25	-0.5(10)
O3	-	C3	-	C4	-	O4	-78.9(5)		C23	-	C24	-	C25	-	C26	-0.1(9)
C2	-	C3	-	C4	-	O4	159.9(4)		C24	-	C25	-	C26	-	C21	1.4(9)
O3	-	C3	-	C4	-	C5	165.3(4)		C22	-	C21	-	C26	-	C25	-2.2(8)
C2	-	C3	-	C4	-	C5	44.2(6)		C20	-	C21	-	C26	-	C25	175.8(5)
C1	-	O1	-	C5	-	C6	-166.8(4)		C3	-	O3	-	C27	-	C28	-177.6(4)
C1	-	O1	-	C5	-	C4	66.4(5)		O3	-	C27	-	C28	-	C33	83.8(6)
O4	-	C4	-	C5	-	O1	-175.2(4)		O3	-	C27	-	C28	-	C29	-95.9(6)
C3	-	C4	-	C5	-	O1	-54.5(6)		C33	-	C28	-	C29	-	C30	0.7(9)
O4	-	C4	-	C5	-	C6	61.0(5)		C27	-	C28	-	C29	-	C30	-179.7(5)
C3	-	C4	-	C5	-	C6	-178.3(5)		C28	-	C29	-	C30	-	C31	0.7(9)
C7	-	O5	-	C6	-	C5	-143.2(4)		C29	-	C30	-	C31	-	C32	-1.8(9)
O1	-	C5	-	C6	-	O5	-68.4(5)		C30	-	C31	-	C32	-	C33	1.6(9)
C4	-	C5	-	C6	-	O5	56.3(6)		C29	-	C28	-	C33	-	C32	-0.9(9)
C6	-	O5	-	C7	-	O6	9.8(8)		C27	-	C28	-	C33	-	C32	179.4(6)
C6	-	O5	-	C7	-	N1	-171.5(4)		C31	-	C32	-	C33	-	C28	-0.2(9)
C8	-	N1	-	C7	-	O6	1.8(9)		C4	-	O4	-	C34	-	C35	157.7(4)
C8	-	N1	-	C7	-	O5	-176.9(4)		O4	-	C34	-	C35	-	C36	145.8(5)
C7	-	N1	-	C8	-	C13	-34.5(8)		O4	-	C34	-	C35	-	C40	-36.1(8)
C7	-	N1	-	C8	-	C9	147.6(5)		C40	-	C35	-	C36	-	C37	-1.7(8)
C13	-	C8	-	C9	-	C10	1.1(8)		C34	-	C35	-	C36	-	C37	176.5(5)
N1	-	C8	-	C9	-	C10	179.1(5)		C35	-	C36	-	C37	-	C38	2.4(8)
C8	-	C9	-	C10	-	C11	-0.3(9)		C36	-	C37	-	C38	-	C39	-1.3(9)
C9	-	C10	-	C11	-	C12	-0.4(9)		C37	-	C38	-	C39	-	C40	-0.4(9)
C10	-	C11	-	C12	-	C13	0.5(9)		C36	-	C35	-	C40	-	C39	-0.1(9)
C11	-	C12	-	C13	-	C8	0.3(9)		C34	-	C35	-	C40	-	C39	-178.1(5)
C9	-	C8	-	C13	-	C12	-1.0(8)		C38	-	C39	-	C40	-	C35	1.1(9)

Supplementary Discussion

The crystals produced in the present work were confirmed to be BCPTG crystals from ^{1}H-NMR and X-ray analyses. Each of the following three factors (“wettability”, “laser ablation”, and “non-photochemical laser-induced nucleation (NPLIN)“) may affect the crystallization observed in the present work, however their contributions were denied as follows.

Contribution of wettability

The general method to suppress the wettability on a surface is to apply silyl coating on the surface. The contribution of the wettability was estimated using a silyl-protected quartz glass cell. The silyl coating in the quartz glass cell was applied up to a height of 20 mm from the bottom to suppress wettability. However, 12 hours of irradiation by the 10-fs laser pulse still produced the same needle-shaped single crystals (Supplementary Figure 4) as those shown in Fig. 2. A methanol solution at a concentration of 1.5 mM was used as a sample. Twenty-four times out of forty experiments have deposited crystal. These results deny the wettability-related contribution.

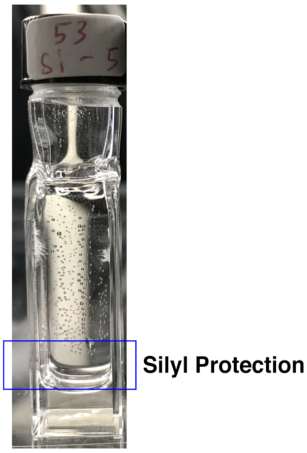

Supplementary Figure 4. **Crystal deposition using a silyl-protected quartz glass cell.** Crystal production from a methanol solution of BCPTG in a silyl-protected quartz glass cell after 12 hours under irradiation with the 10-fs visible laser pulse.

As an additional test, the experiment was repeated in a quartz glass cell with a glass rod pointing downward from the center of the top plastic screw cap and with the tip located at a height 8 mm above the solution surface (Supplementary Figure 5a). The glass rod is isolated from the quartz glass wall by a plastic cap, so the glass rod cannot be affected by the wettability. If the vaporization proceeds by physical phase transformations, the crystal should be deposited not only on the glass cell wall but also on the glass rod. If the crystal is not from vaporization but from wettability, the crystal should not be deposited on the glass rod. The solution in this cell was irradiated using the same laser for 12 hours, and a drop of liquid appeared at the bottom of the glass rod (Supplementary Figure 5b). Further irradiation for 36 hours still did not cause crystal deposits, resulting in the appearance of a liquid drop at the bottom of the glass rod. Upon allowing the liquid droplet to dry, crystals were formed with the shape shown in Supplementary Figure 5c; these crystals were confirmed to consist of BCPTG using ^{1}H-NMR analysis (Supplementary Figure 6).

This experimental result can be explained as follows. Irradiation of the 10-fs visible laser pulse vaporized BCPTG and methanol, which cooled on the glass rod and were solidified and devolatilized, respectively. The deposited BCPTG was solved by devolatilized methanol to be observed as a drop at the bottom of the glass rod. After drying the drop, we obtained a crystal of BCPTG. These results confirm that the wettability of the cell walls is not responsible for the transport of either the BCPTG or the methanol solvent and does not provide the pathway for the observed crystallization.

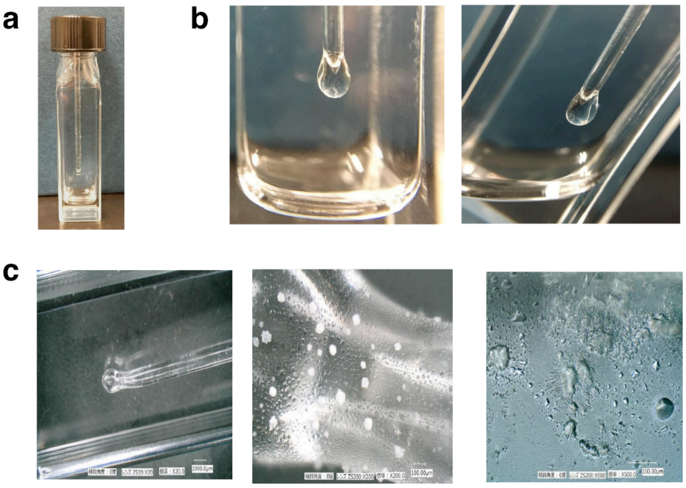

Supplementary Figure 5. **Crystal deposition using a quartz glass cell with a glass rod. a** Photographic image showing the glass rod fixed below the plastic screw cap. **b** Liquid drop on the bottom of the glass rod. **c** Crystals obtained on the glass rod following solvent evaporation.

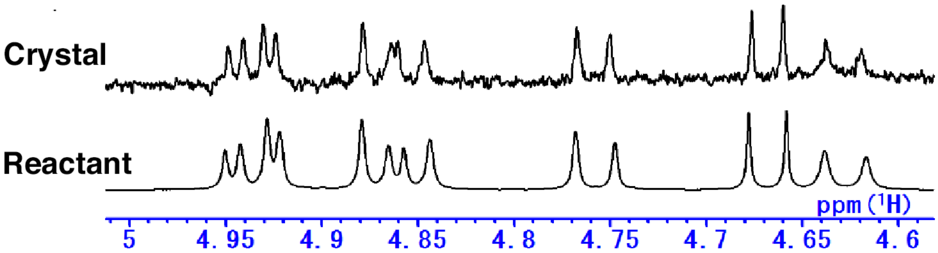

Supplementary Figure 6. ^{1}H NMR spectra of the reactant and the crystals appearing on the glass rod.

Contribution of laser ablation

Single crystal formation via laser ablation can be caused by crystal deposition on the glass cell surface either from solution drop splashed, from aerosol of the splashed solution floating in the glass cell, or gas-evaporated molecule in the glass cell. The contribution of the laser ablation was estimated by increasing the height of the quartz glass cell, by dependence of the depth of the irradiation point from the liquid level, and by the irradiation power dependence. Ablation threshold of organic compounds[3] are reported to be 5-30 mJ cm^{-2}. Thus, the irradiation of the visible 10-fs pulse laser with power of 28 mJ cm^{-2} might have caused the ablation.

For the solution splashed by the laser ablation, its crystallization on the glass cell surface relies on the transport of heavy liquid droplets against gravity within the quartz glass cell and should therefore be dependent on height. To test this hypothesis, a methanol solution of BCPTG was irradiated in a tall glass cell with a height of 100 mm (2.5 times higher than the original quartz glass cell). After irradiating for 12 hours, a comparable deposition morphology of the needle-shaped crystals was formed at the top edge of the tall glass cell (Supplementary Figure 7). It is thought to be not likely that the crystal was formed from the droplet splashed by the laser ablation. In the following, we have considered about the possibility that crystallization on the glass cell surface has occurred from the splashed droplet floating in the glass cell as an aerosol or from evaporated gas molecule.

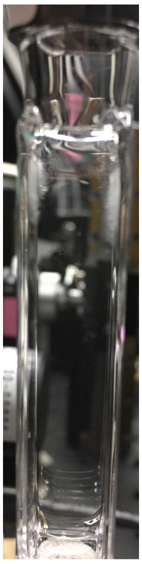

Supplementary Figure 7. Crystal deposition using a quartz glass cell with a height of 100 mm. Methanol solution of BCPTG in a glass cell with a height of 100 mm after 12 hours of laser irradiation.

Under laser ablation, the amount of liquid droplets transported should also depend on the depth of the irradiation point below the solution surface and the irradiation power. It is known that laser abrasion is more efficient when the irradiation is closer to the solution surface and does not occur under the threshold of the irradiation intensity. We therefore repeated the experiment (a) with the depth of the irradiation point varying from just below the solution surface to depths of 0.5, 2.0, and 3.5 mm below the solution surface and (b) with the irradiation power set to 0.5, 0.9, 4, 9, 21, 26, and 28 mJ cm^{-2}. All of these experiments showed no observable difference in the deposition morphology of the needle-shaped crystals. These results again argue against the ablation-related contribution, because the crystal was deposited in the present work with 10% of the threshold power reported in Ref. 3.

Contribution of NPLIN

Effect from possible mechanisms of NPLIN were estimated as below.

At first, we have considered about contribution from optical Kerr effect. There were reports of crystallization by optical Kerr effect using nano second pulse lasers[4,5]. For example, irradiation of an aqueous urea solution by a nanosecond pulse laser (1.06 μm, 20 ns, 0.1 J pulse^{-1}, 5 J cm^{-2}) was reported to align the molecule in the direction of the electric field by the optical Kerr effect, which induced crystallization by increasing the local concentration.[4] We also have tried crystallization of BCPTG focusing a nano second Nd:YAG laser pulse (center wavelength 532 nm, pulse duration 10 ns, pulse energy 1 μJ, pulse area 36 μm^2, 2.6 J cm^{-2}) whose peak power density is comparable with the condition of Ref. 4. We have irradiated the laser pulse over 12 hours repeating five times not finding any deposited crystal, which confirms that the effect of optical Kerr effect is negligible for the visible pulse irradiation on this sample.

Second, cavitation bubbles were considered. Threshold to form cavitation bubbles was reported to be 0.4 - 4 μJ pulse^{-1} (80 - 400 J cm^{-2}) for 200-fs laser pulse and 0.9 - 8 μJ pulse^{-1} (180 - 1620 J cm^{-2}) for 1800-fs laser pulse[6]. The visible 10-fs laser pulse used in the present work has 28 nJ pulse^{-1} (28 mJ cm^{-2}) being much lower than the threshold is thought to be not causing cavitation bubble.

Photon pressure can be described by electro-magnetic theory as gradient force, scattering force absorption force, and resonance force. Photon pressure can trigger crystallization by trapping particle in liquid, liquid-liquid boundary, gas-liquid boundary, solid-liquid boundary. Thus, this trapping phenomena is called as optical trapping. When this method is applied for nanometer-micrometer size particle, the trapped particle can be moved in space by shifting the irradiation point as if the photon pressure trapping the particle can work as a (laser) tweezer. The effect of the photon pressure is most dominant at the focusing spot of the laser being proportional to the light power density. In the present work, the crystal deposition was found not

in the solution at the laser irradiation spot but from the gas phase at 30 mm above the solution level (32 mm above the light irradiation spot). Therefore, the crystal was thought to be deposited not by the standard trapping process by the photon pressure. Note that coherent molecular vibrational excitation is caused by interaction between electric field of the light and polarized chemical bond, which can be affected by the photon pressure.

Contribution from high order multiphoton absorption was denied from the measured transient absorption signal as follows. If the sample is excited to the electronic excited state by the multiphoton absorption process, transient absorption signal is expected to show positive or negative sign reflecting induced absorption or stimulated emission, respectively, from the electronic excited state[7,8]. However, the measured transient absorption signal was oscillating around zero (see Fig. 3) indicating that the measured signal reflects the dynamics of the electronic ground state[7-9], which denies the contribution from the high order multiphpton absorption.

Supplementary Table 5. Polarization dependence using quartz glass rectangular cell with silyl protection.

	a	b	c	d	e	f	g	h	i
Horizontal polarization	√	√	√	-	√	√	√	√	-
Vertical polarization	√	-	√	√	√	√	√	√	-

Supplementary Table 6. Polarization dependence using NMR quartz glass tube.

	j	k	l	m	n	o
Horizontal polarization	√	√	√	-	√	-
Vertical polarization	√	-	-	√	√	√

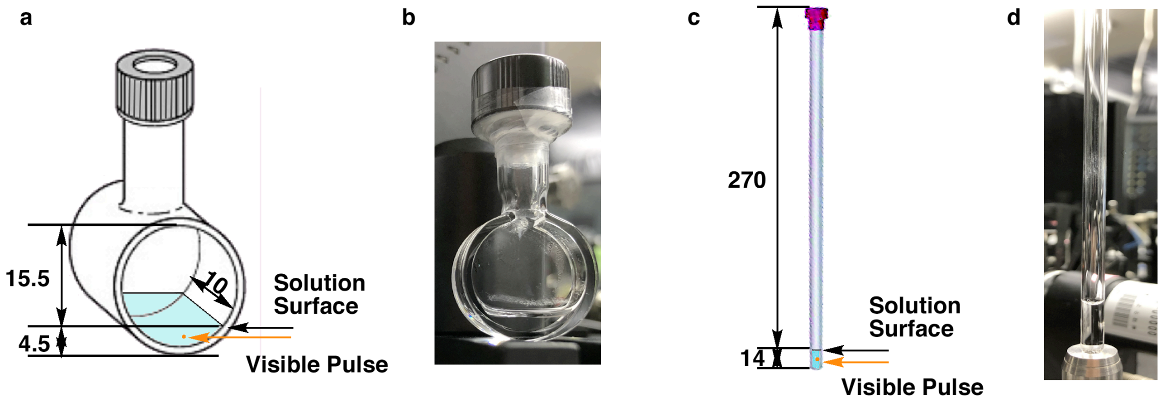

Supplementary Figure 8. Crystal deposition using a cylindrical quartz cell and an NMR quartz glass tube. Schematic figures (**a, c**) and photographs (**b, d**) of a cylindrical quartz cell (**a, b**) and a NMR quartz glass tube (**c, d**) used for crystal production from a methanol solution of BCPTG. The photographs show the solution after 12 hours of irradiation with the 10-fs visible laser pulse. Unit of numbers in the schematic diagram is mm. In the cylindrical quartz cell, the solution was filled to a height of 4.5 mm from the bottom of the container, and laser pulse was irradiated on the solution approximately 2 mm below the solution surface. In the NMR tube, the solution was filled to a height of 14 mm from the bottom of the container, and laser pulse was irradiated on the solution approximately 2 mm below the solution surface.

Supplementary Methods

Sample Synthesis

Dried D-glucose (Sigma-Aldrich, G8270) was fully acetylated (Ac) with acetic anhydride and sodium acetate to produce compound **2**. The boron trifluoride-diethylether complex was added to compound **2** as a catalyst for the selective production of phenylthio (SPh) glycoside (compound **3**). The Ac groups were removed from compound **3** under Zemplén conditions, and then the 6-position was selectively protected by a triphenylmethyl (Tr) group to obtain compound **5**. The hydroxyl groups in **5** were protected by benzyl (Bn) groups to give compound **6**, and the Tr group was removed using a 70% acetic acid aqueous solution to yield compound **7**. The final sugar substrate (compound **1**, denoted as BCPTG in the main paper) was synthesized by adding the *N*-phenylcarbamoyl (Car) group to compound **7** using phenyl isocyanate and pyridine.

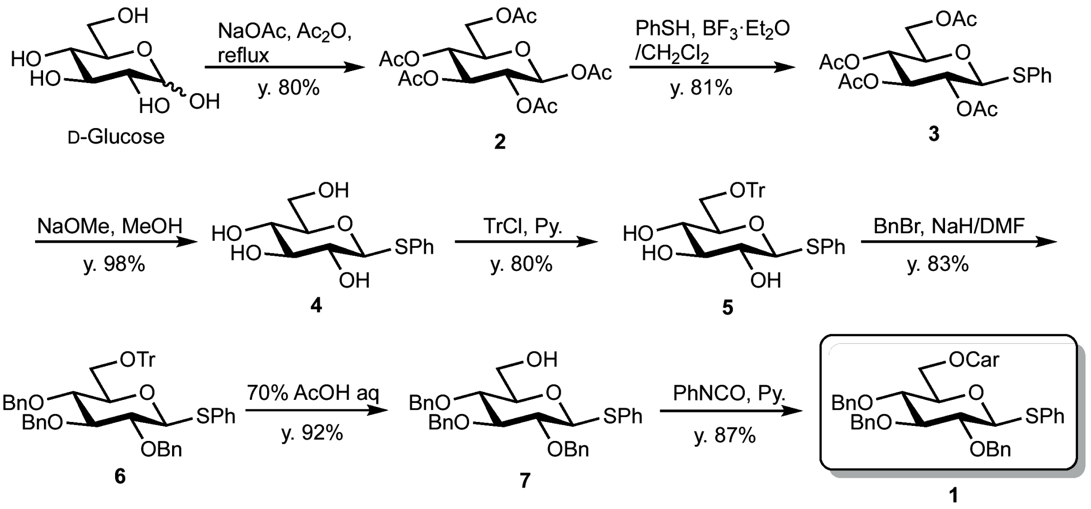

Compound 2: 1,2,3,4,6-Penta-*O*-acetyl-β-D-glucopyranose[10]. To a gently refluxed solution of Ac_2O (280 mL, 3.0 mol) containing anhydrous sodium acetate (12.0 g, 0.14 mol) was slowly added powdered D-glucose (50.0 g, 0.28 mmol) over a period of 15 min. After the mixture was heated to reflux for more than 10 min, the mixture was cooled to room temperature. The reaction was then quenched by the addition of ice and stirred at 0 °C for 1 h. During this treatment, compound **2** was precipitated as a brownish white solid (crude penta-*O*-acetate). The precipitate was filtered and washed with water until the odor of acetic acid disappeared. The crude product was purified by recrystallization with EtOH to afford **2** (86.6 g, 80% yield). $[\alpha]_D^{25}$ +4° (*c* 1.0, $CHCl_3$); mp 132-135 °C (colorless prisms); IR (KBr disk) ν 1755, 1740 cm^{-1}; ^1H NMR (600 MHz, $CDCl_3$) δ 5.72 (1H, d, $J_{1,2}$ = 8.4 Hz, H-1), 5.26 (1H, dd, $J_{2,3}$ = 10.0 Hz, H-2), 5.14 (1H, dd, $J_{3,4}$ = 9.6 Hz, H-3), 5.13 (1H, dd, H-4), 4.29 (1H, dd, $J_{6,6'}$ = 12.6 Hz, H-6), 4.12 (1H, dd, H-6'), 3.84 (1H, ddd, $J_{5,4}$ = 10.2 Hz, $J_{5,6}$ = 4.6 Hz, $J_{5,6'}$ = 1.8 Hz, H-5), 2.11, 2.09, 2.04, 2.04, 2.02 (15H, each s, $COCH_3$); ^{13}C NMR (150 MHz, $CDCl_3$) δ 170.6, 170.1, 169.4, 169.2, 168.9, 91.7, 72.8, 72.7, 70.2, 67.7, 61.4, 20.8, 20.7, 20.5, 20.5. Anal calcd for $C_{16}H_{22}O_{11}$; C, 49.23; H, 5.68, found C, 48.77; 5.75.

Compound 3 (APTG): 2,3,4,6-Tetra-*O*-acetyl-1-phenylthio-β-D-glucopyranoside[11]. Under an argon atmosphere, penta-*O*-acetate **2** (50.0 g, 128 mmol) was dissolved in anhydrous CH_2Cl_2 (500 mL), and BF_3•Et_2O (21.0 mL, 166 mmol) was added at 0 °C. At –5 °C, thiophenol (21.0 mL, 192 mmol) was added dropwise, and the reaction mixture was allowed to warm to room temperature. After complete consumption of the starting penta-*O*-acetate **2**, a saturated sodium bicarbonate solution was added until all the BF•Et_2O was hydrolyzed. The organic layer was washed with water and a saturated sodium bicarbonate solution three times. The combined organic layer was dried over $MgSO_4$, filtered, and concentrated *in vacuo*. The resulting residue was purified on a column of silica gel with hexane-EtOAc (3:1-2:1 v/v) to give β-thioglycoside **3** (45.6 g, 81%

yield); mp 115-117 °C (colorless needles from hexane-EtOAc); IR (KBr disk) ν 1748 cm^{-1}; ^{1}H NMR ($CDCl_3$, 600 MHz) δ 7.51-7.27 (5H, m, Ph*H*), 5.23 (1H, dd, $J_{3,2}$ = 9.0 Hz, $J_{3,4}$ = 9.6 Hz, H-3), 5.04 (1H, dd, $J_{1,2}$ = 10.2 Hz, H-2), 4.98 (1H, dd, $J_{4,5}$ = 10.2 Hz, H-4), 4.71 (1H, d, H-1), 4.23 (1H, dd, $J_{6,6'}$ = 12.3 Hz, $J_{6,5}$ = 5.4 Hz, H-6), 4.18 (1H, dd, $J_{6',5}$ = 2.4 Hz, H-6'), 3.73 (1H, ddd, H-5), 2.08, 2.07, 2.01, 1.98 (12H, each s, $COCH_3$); ^{13}C NMR ($CDCl_3$, 125 MHz) δ 170.3, 170.1, 169.2, 169.0, 133.7, 128.7, 85.9, 76.2, 74.4, 70.3, 68.5, 62.3, 20.9, 20.8.

Compound 4: 1-Phenylthio-β-D-glucopyranoside[12]. A sodium methoxide-methanol solution (5 mL of a 1 M solution) was added to a solution of tetra-*O*-acetate **3** (10.0 g, 22.7 mmol) in methanol (200 mL), and the mixture was stirred at room temperature for 1 h. After complete consumption of starting tetra-*O*-acetate **3**, as determined by thin layer chromatography (TLC) with hexane-EtOAc (2:1 v/v), the mixture was neutralized using an ion-exchange resin (Dowex 50W-X8, H^+ form), filtered, and concentrated in vacuo to give **4** (6.05 g, 98% yield), which was used without further purification; mp 103-105 °C; IR (KBr disk) ν 3405, 1583, 1480 cm^{-1}; ^{1}H NMR (500 MHz, CD_3OD) δ 7.56 (m, 2H), 7.26 (m, 3H), 4.59 (1H, d, $J_{1,2}$ = 10.0 Hz), 3.86 (1H, dd, $J_{6,6'}$ = 12.0 Hz, $J_{6,5}$ = 1.8 Hz, H-6), 3.67 (1H, dd, $J_{6',5}$ = 5.5 Hz, H-6'), 3.38 (1H, dd, $J_{2,3}$ = 9.0 Hz, H-2), 3.31 (2H, m, H-4 and 5), 3.21 (dd, $J_{3,4}$ = 9.0 Hz, H-3); ^{13}C NMR (125 MHz, $CDCl_3$) δ 135.3, 132.8, 129.7, 128.3, 89.4, 82.1, 79.7, 73.8, 71.4, 62.7.

Compound 6: 2,3,4-Tri-*O*-benzyl-1-phenylthio-6-*O*-triphenylmethyl-β-D-glucopyranoside[13]. A solution of **4** (1.0 g, 3.67 mmol) and triphenylmethyl chloride (1.33 g, 4.77 mmol) in pyridine-CH_2Cl_2 (50 mL, 1:1 v/v) was stirred at room temperature for 24 h. After complete consumption of starting compound **4**, as determined by TLC with $CHCl_3$-MeOH (8:1 v/v), the reaction mixture was poured into brine and extracted with $CHCl_3$.

The combined organic layer was washed with water, dried over $MgSO_4$, and concentrated in vacuo. The resulting residue was purified on a column of silica gel with EtOAc to give 1-phenylthio-6-*O*-triphenylmethyl-β-D-glucopyranoside **5** (1.51 g, 80% yield). Benzyl bromide (BnBr) (0.83 mL, 7.0 mmol) was added dropwise to a mixture of **5** (1.0 g, 1.94 mmol) and NaH (312 mg, 7.8 mmol dispersed in 60% mineral oil) in dry DMF (30 mL) at 0 °C. The resulting mixture was stirred at room temperature for 5 h. Excess BnBr and NaH were quenched with Et_3N (0.5 mL) and methanol (1 mL), poured into the brine, and extracted with EtOAc. The combined organic layer was washed with brine, dried over $MgSO_4$, and concentrated in vacuo. The resulting residue was purified on a column of silica gel with hexane-EtOAc (3:1 v/v) to give **6** as a yellow syrup (1.27 g, 83% yield). ^{1}H NMR (500 MHz, $CDCl_3$) δ 7.16-7.52 (35H, m, Ph*H*), 4.56-4.93 (6H, m, OCH_2Ph), 4.31 (1H, d, $J_{1,2}$ = 10.2 Hz, H-1), 3.74 (1H, dd, $J_{3,4}$ = 9.5 Hz, H-3), 3.58-3.69 (3H, m, H-6, H-6', and H-4), 3.45 (1H, dd, $J_{2,3}$ = 9.5 Hz, H-2), 3.26 (1H, m, H-5); ^{13}C NMR (125 MHz, $CDCl_3$) δ 138.3, 138.1, 137.6, 133.8, 131.9, 128.9, 128.8, 128.5, 128.4, 128.2, 128.1, 127.8, 127.6, 127.4, 126.9, 87.3, 86.8, 86.5, 80.8, 78.8, 76.7, 76.0, 75.4, 75.0, 72.1, 62.4.

Compiund 5: 6-*O*-triphenylmethyl-1-phenylthio-β-D-glucopyranoside[13]. ^{1}H NMR (600 MHz, CD_3OD) δ 7.68-7.66, 7.47-7.46, 7.28-7.20 (20H, m, Ph*H*), , 4.67 (1H, d, $J_{1,2}$ = 9.6 Hz, H-1), 3.46 (1H, ddd, $J_{5,6a}$ = 1.8 Hz, $J_{5,6b}$ = 6.8 Hz, $J_{5,4}$ = 9.8 Hz, H-5), 3.42 (1H, dd, $J_{6a,6b}$ = 10.0 Hz, H-6a), 3.33 (1H, dd, $J_{4,3}$ = 8.9 Hz, H-4), 3.25 (1H, dd, $J_{2,3}$ = 9.6 Hz, H-3), 3.25 (1H, dd, H-6b), 3.23 (1H, dd, $J_{2,3}$ = 9.2 Hz, H-2); ^{13}C NMR (150 MHz, CD_3OD) δ 145.5, 135.5, 132.6, 130.0, 130.0, 128.7, 128.6, 128.2, 128.0, 89.1, 87.8, 80.9, 79.9, 73.9, 71.7, 65.1.

Compound 1 (BCPTG): 2,3,4-Tri-*O*-benzyl-6-*O*-(*N*-phenylcarbamoyl)-1-phenylthio-β-D-glucopyranoside[14]. A mixture of **6** (1.01 g, 1.29 mmol) in a 70% acetic acid solution (50 mL) was stirred at room temperature for 12 h. After complete consumption of **6**, as determined by TLC with hexane-EtOAc (2:1 v/v), the mixture was concentrated in vacuo. The resulting residue was purified on a column of silica gel with hexane-EtOAc (2:1-1:1 v/v) to give **7** (2,3,4-tri-*O*-benzyl-6-hydroxy-1-phenylthio-*β*-D- glucopyranoside, BHPTG) (642 mg, 92% yield). Next, phenyl isocyanate (0.12 mL, 1.1 mmol) was added to a solution of **7** (501 mg, 0.923 mmol) in dry pyridine (20 mL) at 0 °C, and the mixture was stirred for 1 h. After complete consumption of the starting compound, as determined by TLC with hexane-EtOAc (2:1 v/v), the mixture was evaporated in vacuo. The resulting crude crystal was purified by recrystallization with hexane-EtOH to give **1** (532 mg, 87% yield). $[\alpha]_D^{25}$ +8.9 ° (*c* 1.51, $CHCl_3$); mp 131-132 °C (colorless needles from hexane-EtOH); IR (KBr disk) ν 3369 cm^{-1} (NH), 1703 cm^{-1} (C=O); ^{1}H NMR (600 MHz, $CDCl_3$) δ 7.55-7.06 (25H, m, Ph*H*), 6.55 (1H, br s, CON*H*), 4.94, 4.87 (2H, each d, J = 10.8 Hz, OCH_2Ph), 4.93, 4.75 (2H, each d, J = 10.2 Hz, OCH_2Ph), 4.85, 4.62 (2H, each d, J = 10.2 Hz, OCH_2Ph), 4.67 (1H, d, $J_{1,2}$ = 10.2 Hz, H-1), 4.44 (1H, br d, $J_{6,6'}$ = 12.0 Hz, H-6), 4.34 (1H, m, H-6'), 3.74 (1H, br dd, $J_{5,4}$ = 9.0 Hz, $J_{5,6}$ = 9.0 Hz, H-5), 3.57-3.53 (2H, m, H-3 and H-4), 3.50 (1H, dd, $J_{2,3}$ = 9.0 Hz, H-2); ^{13}C NMR (150 MHz, $CDCl_3$) δ 138.2, 137.8, 137.5, 133.3, 132.4, 129.1, 128.8, 128.5, 128.5, 128.5, 128.4, 128.3, 128.2, 127.8, 123.5, 118.5, 87.6, 86.7, 81.0, 77.2, 77.1, 75.8, 75.5, 75.0; Anal. calcd for $C_{40}H_{39}NO_6S$ (661.81) C, 72.59; H, 5.94; N, 2.12, found C, 72.21; H, 5.60; N, 1.99; HRMS (ESI-TOF) calcd for $C_{40}H_{39}NO_6S$ *m/z* $[M+Na]^+$ 684.2396, found 684.2376.

Additional data: cif

a

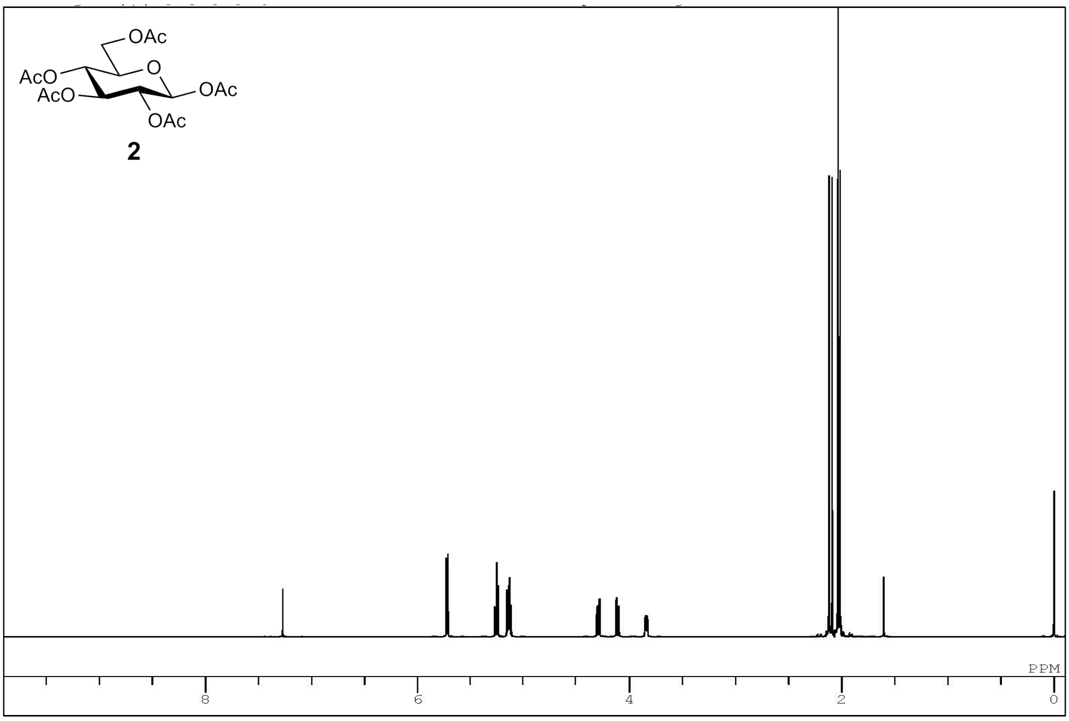

b

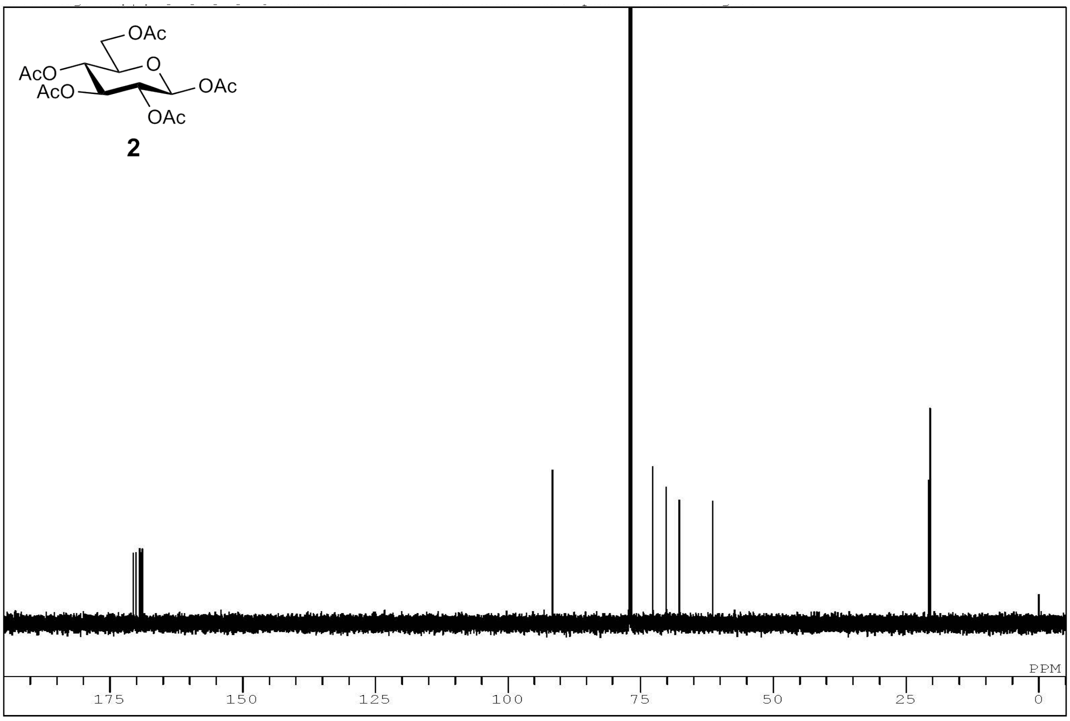

Supplementary Figure 9. **NMR spectra of 1,2,3,4,6-penta-*O*-acetyl-*β*-D-glucopyranose (compound 2).**
a ^{1}H NMR and **b** ^{13}C NMR.

a

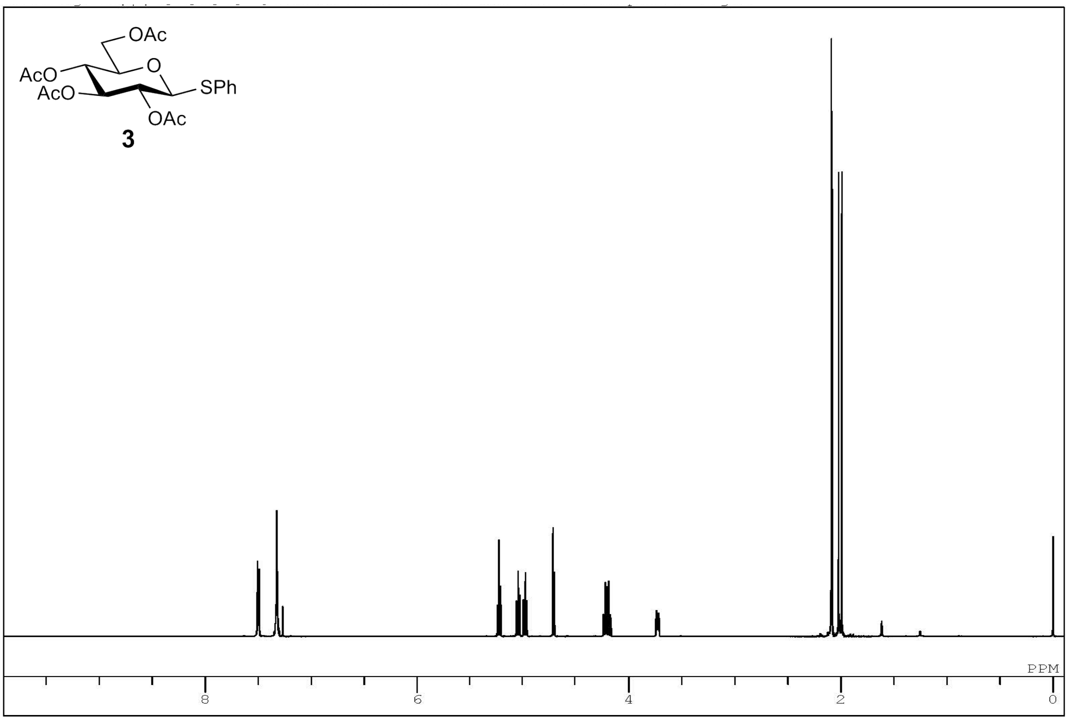

b

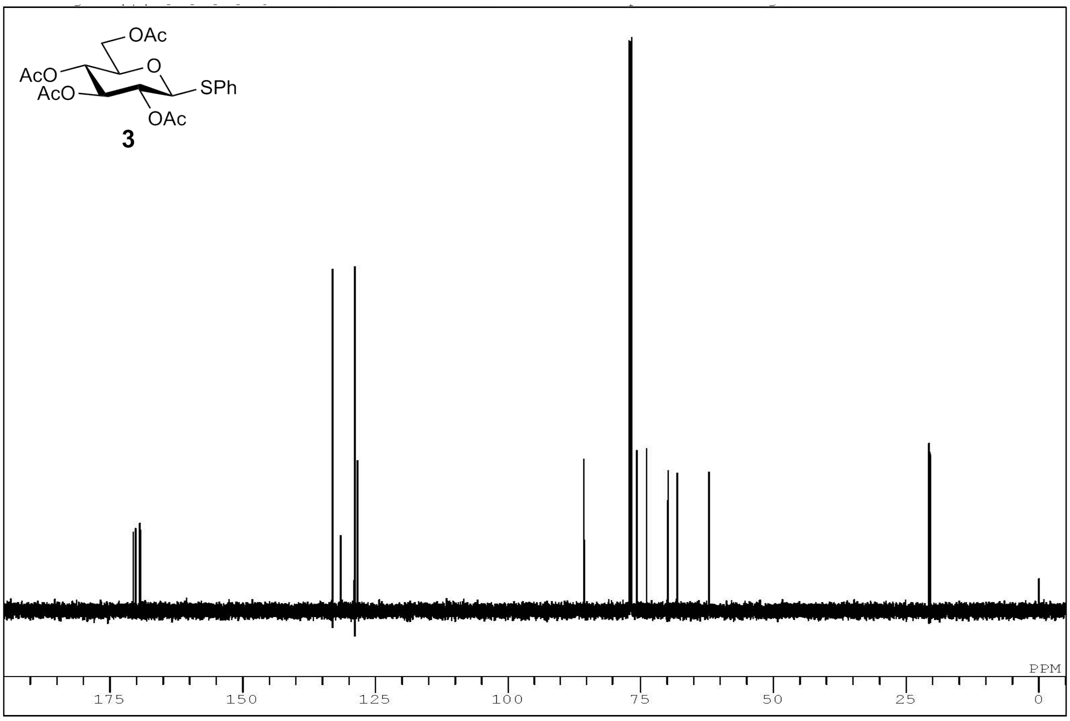

Supplementary Figure 10. **NMR spectra of 2,3,4,6-tetra-*O*-acetyl-1-phenylthio-*β*-D-glucopyranoside (compound 3). a** ^{1}H NMR and **b** ^{13}C NMR.

a

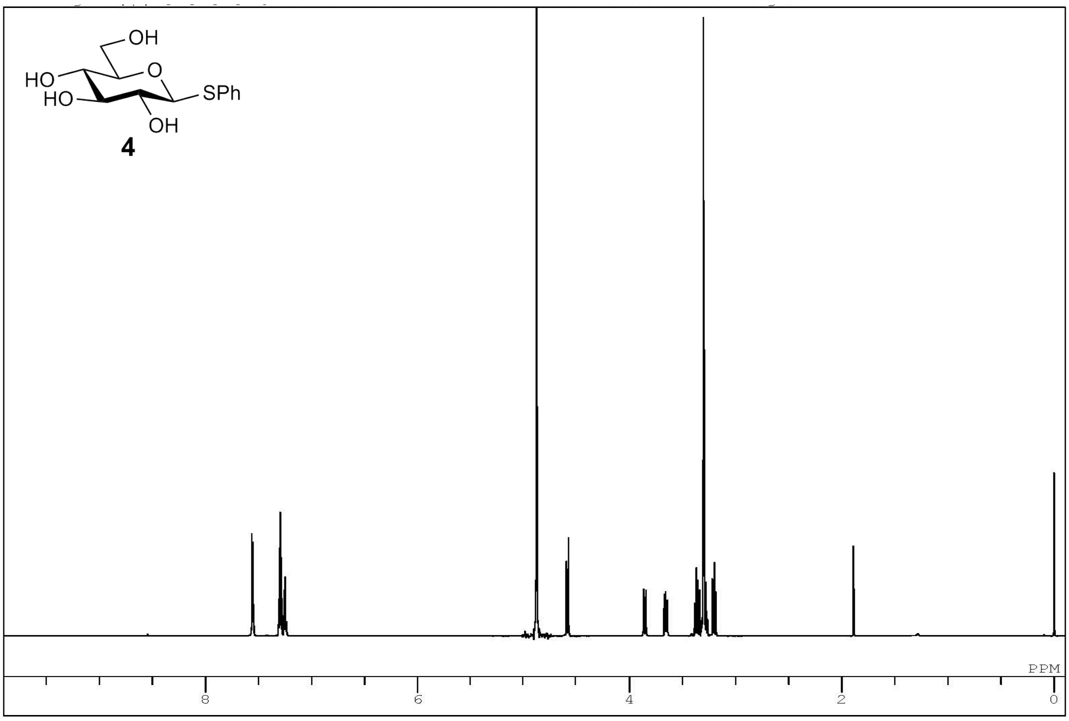

b

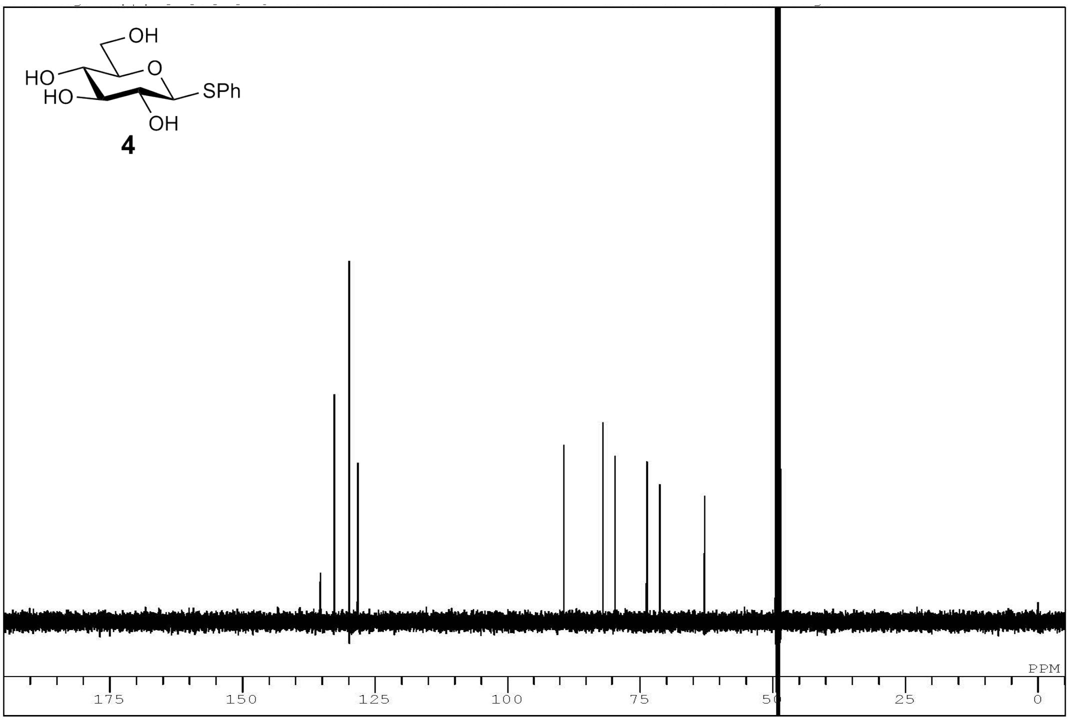

Supplementary Figure 11. **NMR spectra of 1-phenylthio-*β*-D-glucopyranoside (compound 4).** **a** ^{1}H NMR and **b** ^{13}C NMR.

a

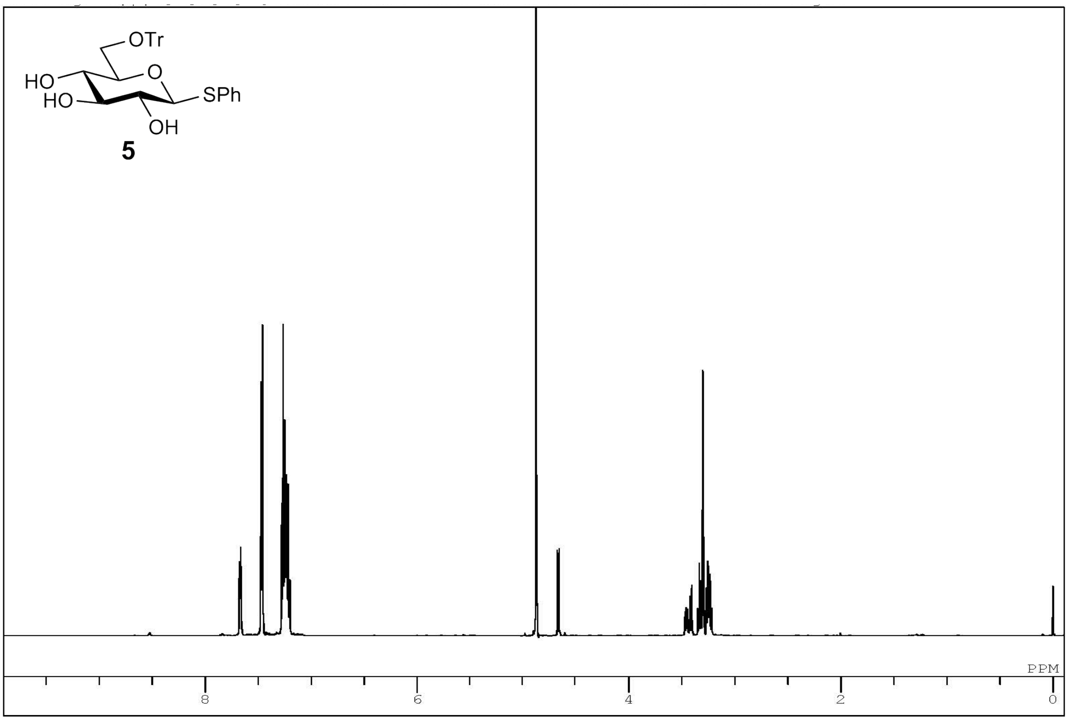

b

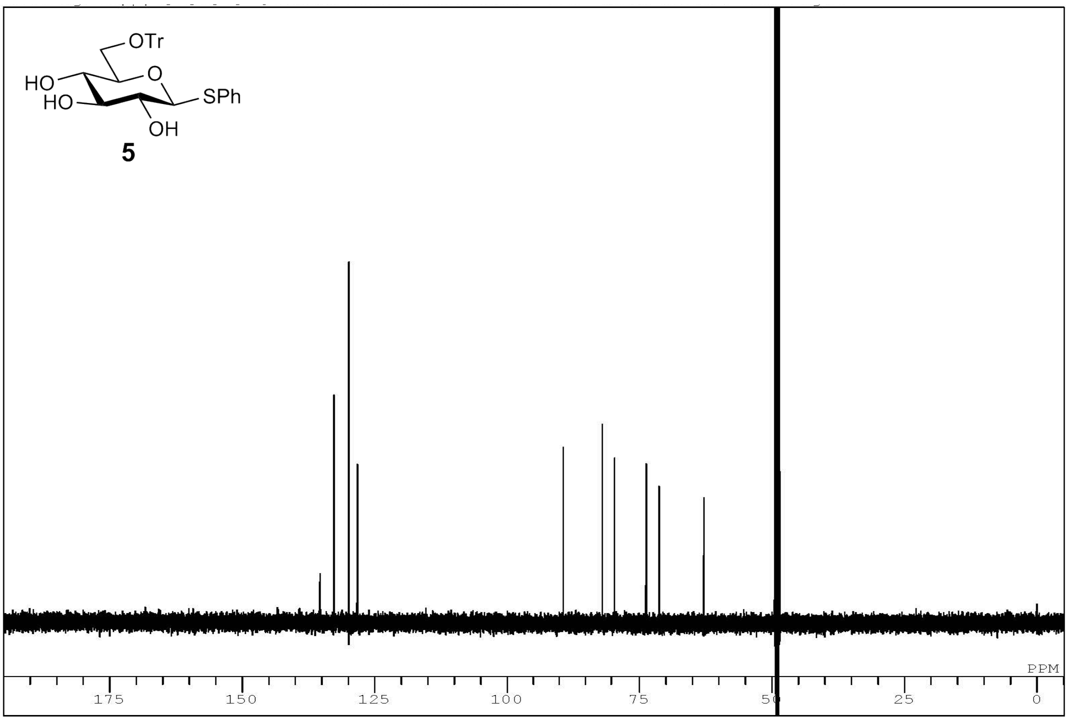

Supplementary Figure 12. NMR spectra of 6-*O*-triphenylmethyl-1-phenylthio-*β*-D-glucopyranoside (compound 5). **a** ^{1}H NMR and **b** ^{13}C NMR.

a

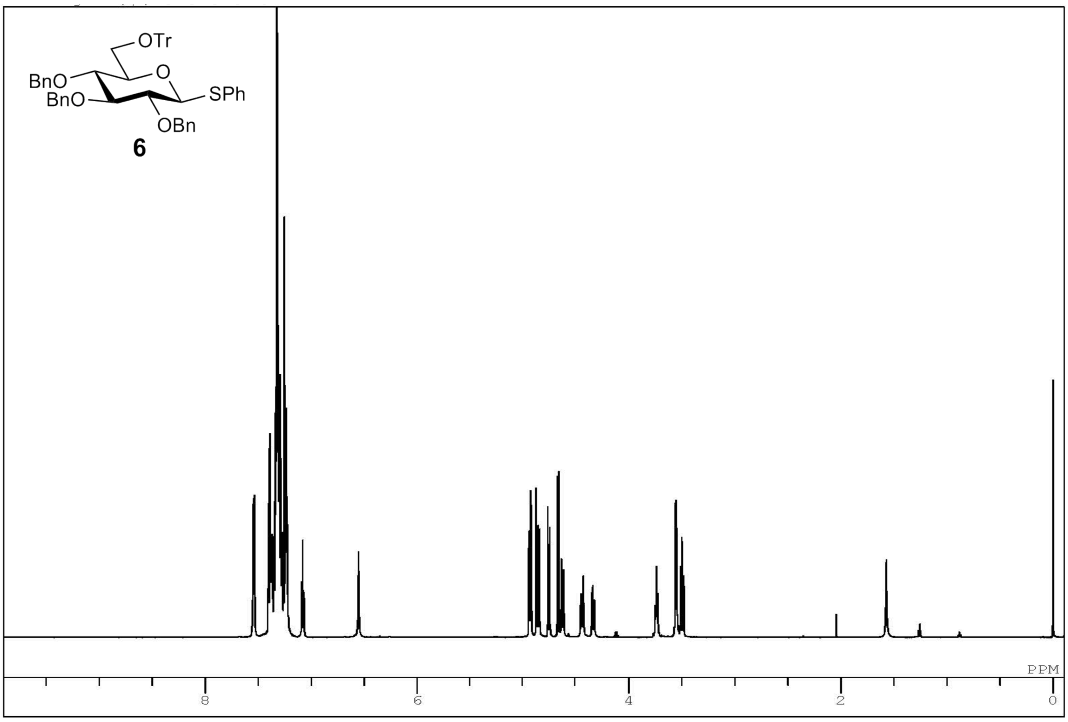

b

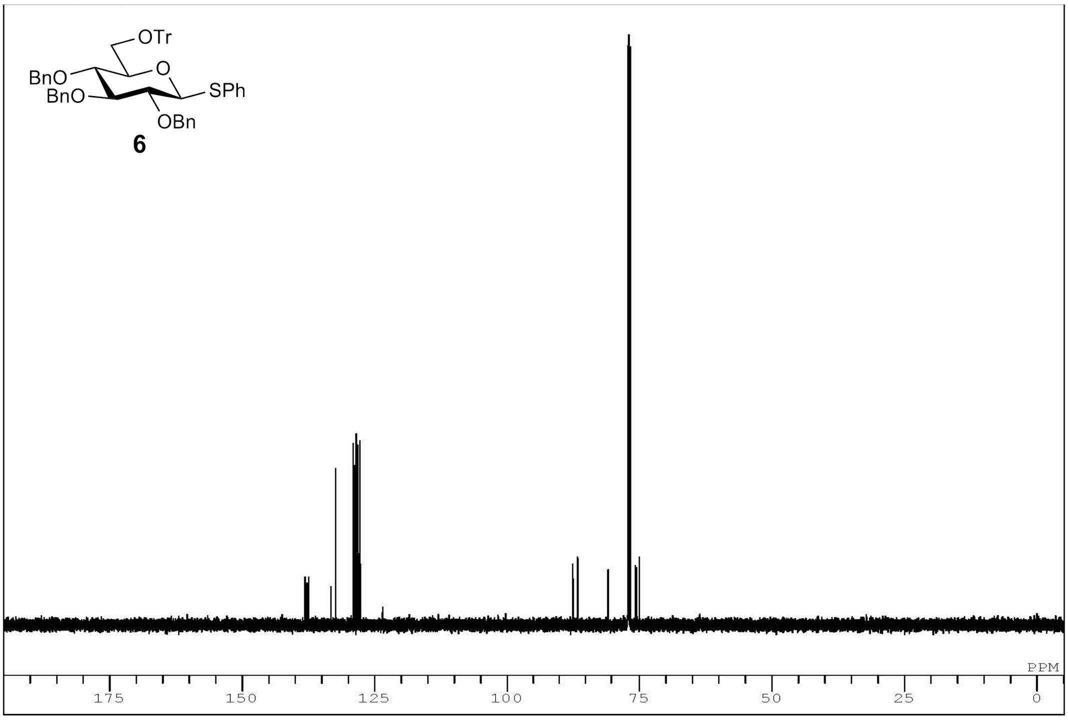

Supplementary Figure 13. **NMR spectra of 2,3,4-tri-*O*-benzyl-1-phenylthio-6-*O*-triphenylmethyl-*β*-D-glucopyranoside (compound 6)**. **a** ^{1}H NMR and **b** ^{13}C NMR.

a

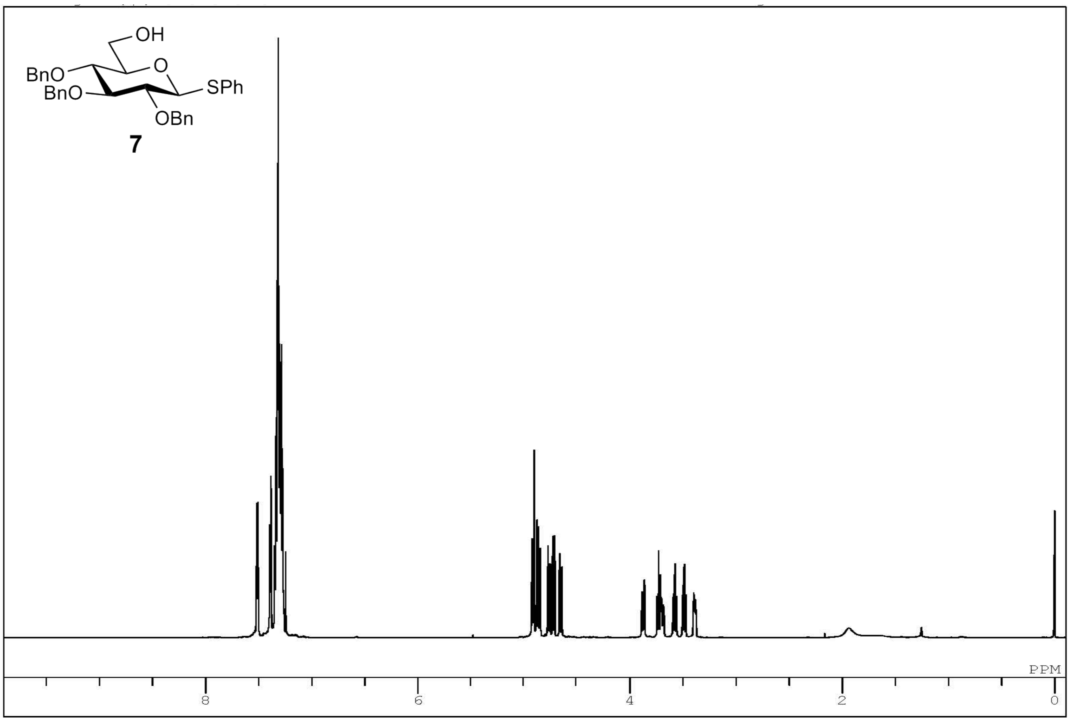

b

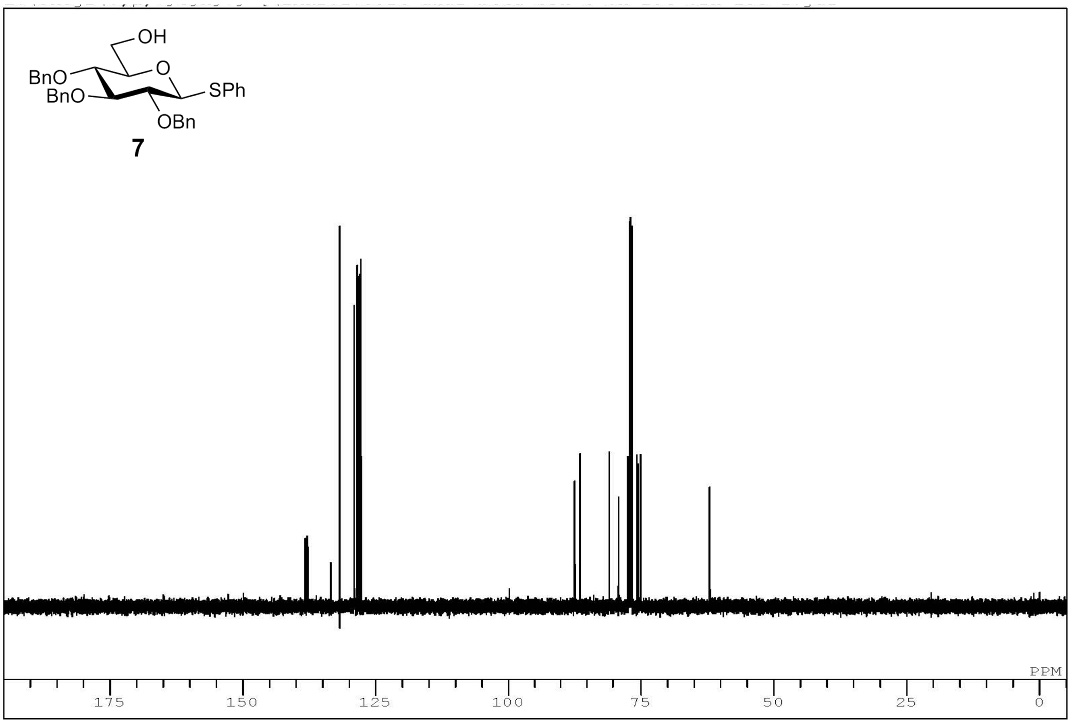

Supplementary Figure 14. **NMR spectra of 2,3,4-tri-*O*-benzyl-6-hydroxy-1-phenylthio-*β*-D-glucopyranoside (BHPTG, compound 7). a** ^{1}H NMR and **b** ^{13}C NMR.

a

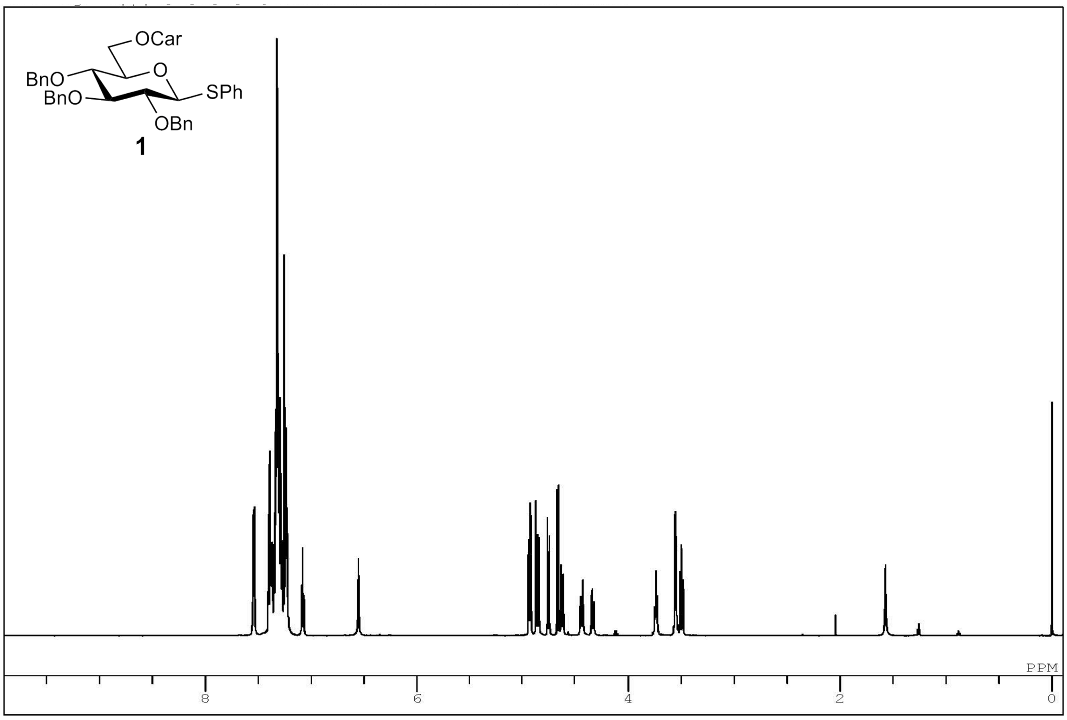

b

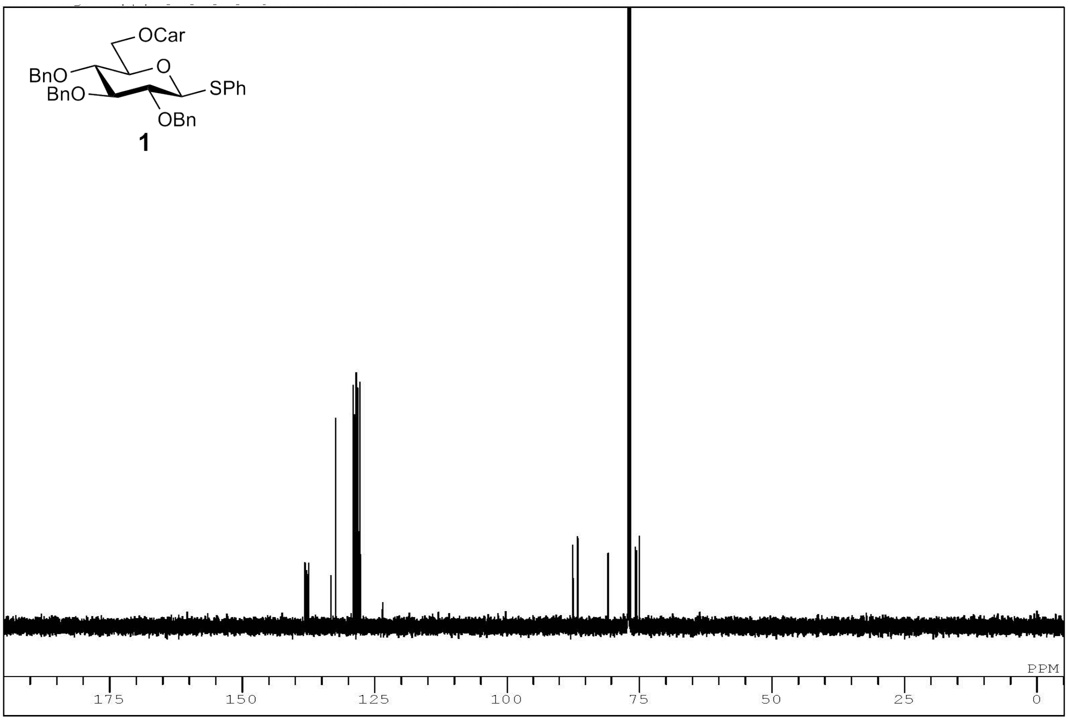

Supplementary Figure 15. **NMR spectra of 2,3,4-tri-*O*-benzyl-6-*O*-(*N*-phenylcarbamoyl)-1-phenylthio-*β*-D-glucopyranoside (BCPTG, compound 1). a** ^{1}H NMR and **b** ^{13}C NMR.

Physical Measurements

The uncorrected melting points were measured using a micro-melting point apparatus (Yanaco, MP-J3). The IR spectra were recorded using an FT-IR spectrometer (JASCO, FT/IR-4200) with KBr pellets. The ^{1}H and ^{13}C NMR spectra were measured with NMR spectrometers (JEOL, JNM-ECA-500 and 600) in chloroform-d, dimethylsulfoxide-d6 or methanol-d3 referenced to tetramethylsilane (0.00 ppm). The specific rotations were measured in a 0.5 dm tube using a polarimeter (JASCO, P-1020) in chloroform or methanol. The mass spectra were obtained by a mass spectrometer (JEOL, JMS-T100CS). Elemental analysis was performed with an elemental analyzer (Perkin-Elmer, 2400II). The assignments of various NMR spectra were assisted by homonuclear ($^{1}H/^{1}H$) correlation spectroscopy (COSY), nuclear Overhauser and exchange spectroscopy (NOESY), nuclear Overhauser effect (NOE), and/or heteronuclear ($^{1}H/^{13}C$) correlation spectroscopy (HETCOR) experiments. Column chromatography was performed on silica gel (Silica gel 60, 70-230 mesh; silica gel 60N, spherical, neutral, 70-230 mesh). TLC on silica gel 60F254 was used to monitor the reactions and to certify the purity of the reaction products by charring after spraying with a phosphomolybdic acid ethanol-H_2SO_4 solution and a cerium ammonium molybdate water-H_2SO_4 solution. The Raman spectra of the samples were obtained using a Raman spectrometer (JASCO, RMP-320). The stationary absorption spectra of the samples were observed using a spectrometer (JASCO, V-650). Movies of the crystal growth process were recorded by a digital microscope (Keyence Corp., VH5500).

The single crystals of BCPTG, formed by sublimation on the wall of the glass cell, were suitable for X-ray analysis. The colorless crystals were coated with paraffin oil, mounted with Micromount (Mitegen), and then immediately cooled at 93 K in cool nitrogen gas. All measurements were obtained on a VariMax Dual (RIGAKU) Saturn724 diffractometer using multilayer mirror monochromated Mo-Ka radiation. Diffraction data were processed using the CrystalClear package[15]. The crystal structure was solved by direct

methods (SIR 2004)[16] and refined by the full-matrix least-squares method on F2 using SHELXL-2014[17] (Supplementary Figure 3 and Supplementary Tables 1-4). The positions of the hydrogen atoms in water molecules were refined using isotropic displacement parameters that restrained the bond lengths. Selected crystal data and structure refinement details are presented in Table S1. All H atoms were positioned geometrically and treated as traveling on their parent atoms. The CCDC reference number is 1834656.

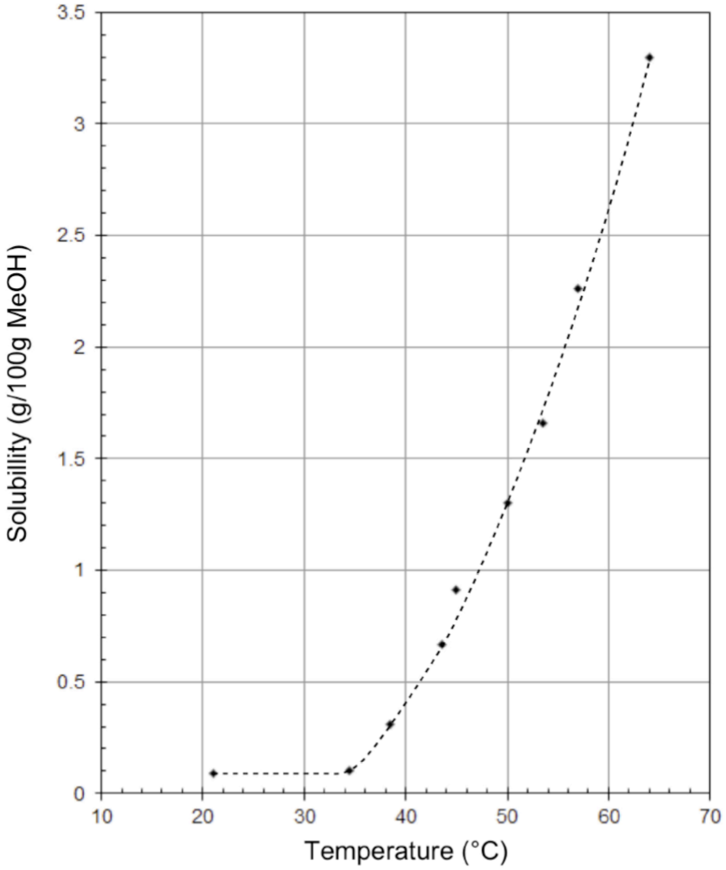

Supplementary Figure 16. **Solubility curve of BCPTG in methanol.**

Dependence on Polarization

In solution, BCPTG molecule has random orientation and the number of the molecule to interact with the laser electric field is thought to be independent on laser polarization. Thus, crystal deposition under reaction by coherent molecular vibration (RCVM) is thought to be independent on laser polarization. The independence was confirmed experimentally by using the experimental setup shown in Supplementary Figure 17.

Sample 1 (2) was irradiated by horizontal (vertical) polarization 10-fs laser at the same time with same experimental parameter for laser spectrum and pulse duration. Supplementary Table 5 shows the experimental results using a silyl-protected quartz glass cell (internal dimensions of 10 × 10 × 40 mm^3). A methanol solution at a concentration of 1.5 mM was used as a sample. Supplementary Table 6 shows the experimental results using an NMR quartz tube. Eleven times out of fifteen experiments have deposited crystal for each of horizontal and vertical polarization.

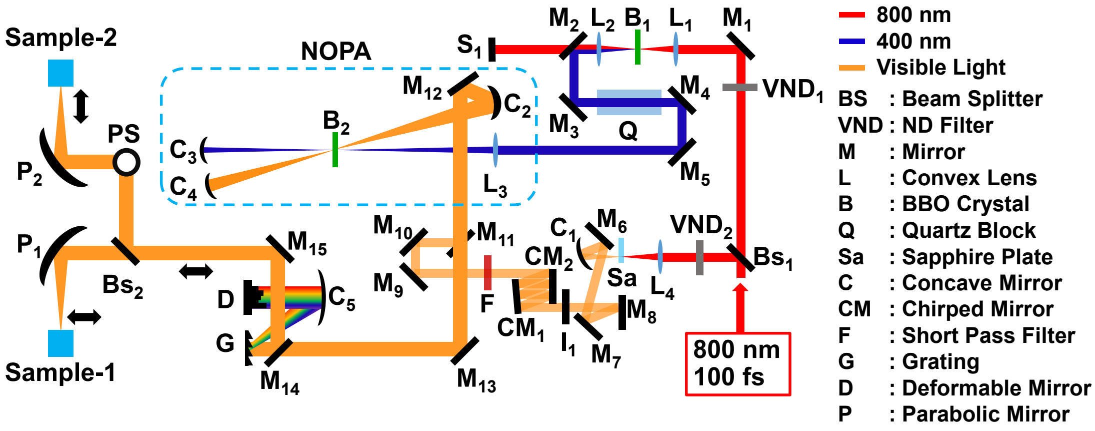

Supplementary Figure 17. Schematic figure of the experimental setup for polarization dependence.

Dependence on the Glass Cell Shape

Supplementary Movie 1 shows that the crystal was deposited at the corner of the quartz glass cell. At a place where a surface is not flat, a molecule has more interaction with the bumpy surface than a flat surface and is easily trapped there. This is thought to be the reason why the nucleation has occurred preferentially at the corner of the glass cell. Dependence on the glass cell shape of the nucleation position was studied as follows.

At first, a quartz cylindrical cell was filled with 200 μl of solution (up to height of 4.5 mm) to be irradiated by the visible 10-fs pulse laser, which deposited crystal at the corner where the top surface is pasted with the side surface of the glass cell (see Supplementary Figure 8a and Supplementary Figure 8b).

Next, a quartz NMR tube (internal dimeter 4 mm, height 270 mm) was filled with 150 μl of solution (up to height of 14 mm), which deposited crystal at 40 mm higher than the solution level when the solution was irradiated by the visible 10-fs pulse laser (see Supplementary Figure 8c and Supplementary Figure 8d). Eight times out of twelve experiments have deposited the crystal. Crystal deposition probability is thought to be lowered because the internal surface of the quartz NMR tube is almost flat.

Thus, we can conclude the crystal deposition by the present method is not dependent on the glass cell shape.

Data for Confirmation of Reproducibility

A quartz glass rectangular cell (wall thickness of 1mm, internal dimensions of 10 × 10 × 40 mm^3) was used to store the sample solution. Methanol solution with concentration of 1.5 mM has deposited crystal for all of twelve times of trial. That with concentration of 1.1 mM deposited crystal for all of eight time of trial. The concentration of 0.8 mM results in crystal deposition for eight out of nine times. The concentration of 0.4 mM results in crystal deposition for three out of five times. In case of the concentration of 0.2 mM, no crystal was deposited for twice of trial.

The 10-fs laser pulse is sensitive to environmental parameters such as room temperature and humidity. The maximum laser intensity of each day has variation because the highest intensity for each day is difference dependent on those environmental factors.

We have added corresponding data in the following Supplementary Tables.

Table 7 Crystal deposition reproducibility for methanol solution in square quartz cell

Table 8 Crystal deposition reproduciblility for methanol solution in silyl protected square quartz cell.

Table 9 Dependence on solvent for square quartz cell.

Table 10 Dependence on structure of quartz cell.

Supplementary Table 7. **Crystal deposition reproducibility for methanol solution of BCPTG in quartz rectangular cell.**

Sample Holder	Solvent	Concentration (mol L^{-1})	Height of Irradiation below the Surface (mm)	Laser Polarization	Irradiation Power (mJ cm^{-2})	Number of Trial	Number of Trial with Crystal	Nucleation Efficiency (%)
Quartz Glass Rectangular Cell	Methanol	1.5	2	Horizontal	29	12	12	100.0
					25			
					25			
					19			
					18			
					5			
					5			
					5			
					5			
					5			
					5			
					5			
		1.1	2	Horizontal	26	8	8	100.0
					21			
					21			
					21			
					9			
					4			
					0.9			
					0.8			
		0.8	2	Horizontal	27	9	8	88.9
					23			
					20			
			0.5	Horizontal	26			
					24			
					22			
			3.5	Horizontal	22			
					17			
					4			
		0.4	2	Horizontal	23	5	3	60.0
					22			
					21			
					20			
					15			
		0.2	2	Horizontal	22	2	0	0.0
					19			

Supplementary Table 8. Crystal deposition reproducibility for methanol solution of BCPTG in quartz rectangular cell with silyl protected.

Sample Holder	Solvent	Concentration (mol L^{-1})	Height of Irradiation below the Surface (mm)	Laser Polarization	Irradiation Power (mJ cm^{-2})	Number of Trial	Number of Trial with Crystal	Nucleation Efficiency (%)
Quartz Glass Rectangular Cell with Silyl Protection	Methanol	1.5	2	Horizontal	28	40	24	60.0
					26			
					24			
					24			
					22 [a]			
					22 [b]			
					21			
					21			
					20 [c]			
					20 [d]			
					20			
					20			
					20			
					20			
					19 [e]			
					19			
					18			
					18			
					18			
					18			
					17 [f]			
					17			
					16 [g]			
					16 [h]			
					16 [i]			
					16			
					15			
				Vertical	28			
					24			
					20 [c]			
					19 [b]			
					18 [d]			
					16 [a]			
					13 [g]			
					13 [h]			
					13 [f]			
					13 [e]			
					12 [i]			
					0.5			
		0.8	2	Horizontal	0.5			

Supplementary Table 9. Dependence on solvent using quartz rectangular cell.

Sample Holder	Solvent	Concentration (mol L^{-1})	Height of Irradiation below the Surface (mm)	Laser Polarization	Irradiation Power (mJ cm^{-2})	Number of Trial	Number of Trial with Crystal	Nucleation Efficiency (%)
Quartz Glass Rectangular Cell	Tetradeutero-methanol	1.5	2	Horizontal	22	2	2	100.0
					14			
	Ethanol	1.5	2	Horizontal	50	4	4	100.0
					23			
		0.8	2	Horizontal	25			
					23			
	2-Propanol	1.0	2	Horizontal	28	2	2	100.0
					21			
	Cyclo-octanol	1.0	2	Horizontal	26	2	0	0.0
					23			
	Acetonitrile	1.5	2	Horizontal	26	2	2	100.0
					25			
	Acetone	1.5	2	Horizontal	34	2	0	0.0
					18			
	Chloroform	1.5	2	Horizontal	21	2	0	0.0
					15			
	Benzene	1.5	2	Horizontal	29	2	0	0.0
					24			
	Toluene	1.5	2	Horizontal	24	2	0	0.0
					22			
	1,4-dioxane	1.5	2	Horizontal	24	2	0	0.0
					15			

Supplementary Table 10. Dependence on structure of quartz cell.

Sample Holder	Solvent	Concentration (mol L^{-1})	Height of Irradiation below the Surface (mm)	Laser Polarization	Irradiation Power (mJ cm^{-2})	Number of Trial	Number of Trial with Crystal	Nucleation Efficiency (%)
Quartz Glass Rectangular Cell with Rod Glass Inside	Methanol	1.5	2	Horizontal	31<br>27<br>25	3	3	100.0
	Ethanol	1.5	2	Horizontal	23	1	1	100.0
Long Quartz Rectangular Cell	Methanol	1.5	2	Horizontal	25<br>20	2	2	100.0
Cylindrical Quartz Glass Cell	Methanol	0.8	2	Horizontal	27	2	1	50.0
					24			
NMR Quartz Glass Tube	Methanol	1.5	2	Horizontal	17 [j]	6	4	66.7
					15 [k]			
					12 [l]			
				Vertical	13 [j]			
					11 [k]			
					10 [l]			
		0.8	2	Horizontal	25 [m]	6	4	66.7
					24 [n]			
					20 [o]			
				Vertical	21 [m]			
					20 [n]			
					20 [o]			

Pump-Probe Measurement

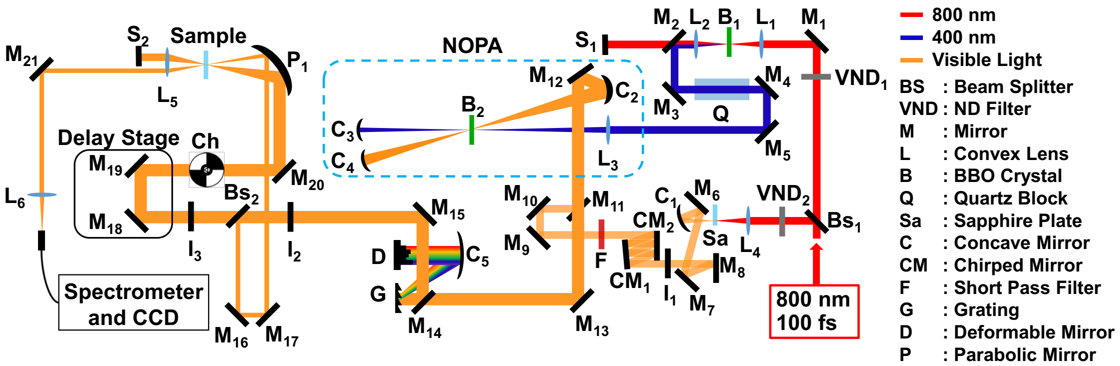

Supplementary Figure 18. **Schematic figure of the pump-probe measurement setup.**

Supplementary References

1. Flack, H. D., Bernardinelli, G. Reporting and evaluating absolute-structure and absolute-configuration determinations. *J. Appl. Cryst.* **33**, 1143–1148 (2000).
2. Parsons, S., Flack, H. D. Wagner, T. Use of intensity quotients and differences in absolute structure refinement. *Acta Cryst.* **B69**, 249–259 (2013).
3. Masuhara, H. Time-Resolved Spectroscopic and Imaging Studies on Laser Ablation of Molecular Systems: From Mechanistic Study to Bio/Nano Applications. *Bull. Chem. Soc. Jpn.* **86**, 755-783 (2013).
4. Garetz, B. A., Aber, J. E., Goddard, N. L., Young, R. G., Myerson, A. S. Nonphotochemical, Polarization-Dependent, Laser-Induced Nucleation in Supersaturated Aqueous Urea Solutions. *Phys. Rev. Lett.* **77** 3475-3476 (1996).
5. Zaccaro, J., Matic, J., Myerson, A. S., Garetz, B. A. Nonphotochemical, Laser-Induced Nucleation of Supersaturated Aqueous Glycine Produces Unexpected y-Polymorph. *Cryst. Growth Des.* **1**, 5-8 (2001).
6. Yoshikawa, H. Y., Murai, R., Maki, S., Kitatani, T. Sugiyama, S., Sazaki, G., Adachi, H., Inoue, T., Matsumura, H., Takano, K., Murakami, S., Sasaki, T., Mori, Y. Laser energy dependence on femtosecond laser-induced nucleation of protein. *Appl. Phys. A* **93**, 911-915 (2008).
7. Iwakura, I. Yabushita, A., Kobayashi, T. Transition states and nonlinear excitations in chloroform observed with a sub-5 fs pulse laser. *J. Am. Chem. Soc.* ***131***, 688-696 (2009).
8. Iwakura, I. The experimental visualization of molecular structural changes during both photochemical and thermal reactions by real-time vibrational spectroscopy. *Phys. Chem. Chem. Phys.* **13**, 5546-5555 (2011).
9. Iwakura, I., Yabushita, A. Development of novel reactions induced by coherent molecular vibrational excitation and direct observation of molecular structural change during "thermal" reactions. *Bull. Chem. Soc. Jpn.* **89**, 296-307 (2016).
10. Michihata, N., Kaneko, Y., Kasai, Y., Tanigawa, K., Hirokane, T., Higasa, S., Yamada, H. High-yield total synthesis of (-)-strictinin through intramolecular coupling of gallates. *J. Org. Chem.* **78**, 4319-4328 (2013).
11. Dieskau, A. P., Plietker, B. A mild iigand-free iron-catalyzed liberation of alcohols from allylcarbonates. *Org. Lett.*, **13**, 5544-5547 (2011).
12. Boulineau, F. P., Wei, A. Mirror-image carbohydrates: synthesis of the unnatural enantiomer of a blood group trisaccharide. *J. Org. Chem.* **69**, 3391-3399 (2004).
13. Agarwal, A., Vankar, Y. D. Selective deprotection of terminal isopropylidene acetals and trityl ethers using $HClO_4$ supported on silica gel. *Carbohydr. Res.* **340**, 1661-1667 (2005).

14. Akai, S., Tanaka, R., Hoshi, H., Sato, K. Selective deprotection method of N-phenylcarbamoyl group. *J. Org. Chem.* **78**, 8802-8808 (2013).
15. Rigaku (2010). CrystalStructure. Version 4.0. Rigaku Corporation, Tokyo, Japan.
16. Burla, M. C., Caliandro, R., Camalli, M., Carrozzini, B., Cascarano, G. L., De Caro, L., Giacovazzo, C., Polidori, G., Spagna, R. SIR2004: an improved tool for crystal structure determination and refinement. *J. Appl. Cryst.* **38**, 381-388 (2005).
17. Sheldrick, G. M. Crystal structure refinement with SHELXL. *Acta Cryst.* **C71**, 3–8 (2015).
