# Supplementary material for: Formation of thioglucoside single crystals by coherent molecular vibrational excitation using a 10-fs laser pulse
Source: Commun Chem. 2020 Mar 17;3:35. doi: 10.1038/s42004-020-0281-6 (PMC9814847; doi:10.1038/s42004-020-0281-6)
Supplement: Supplementary file 2 — Description of Additional Supplementary Files [file 42004_2020_281_MOESM2_ESM.docx]

Description of Additional Supplementary Material files

Name: Supplementary Movie 1

Description: Crystal deposited using irradiation of 10-fs laser pulse

Name: Supplementary Data 1

Description: Crystallographic information file for 2,3,4-tri*-O-*benzyl*-*6*-O-*(N-phenylcarbamoyl)-1-phenylthio-β-D-glucopyranoside (BCPTG)
